# Potassium-competitive acid blockers and proton-pump inhibitors for the healing and maintenance of erosive esophagitis: a comprehensive and updated systematic review with network meta-analysis

**DOI:** 10.1007/s10388-026-01222-2

**Published:** 2026-06-15

**Authors:** Daniel Martin Simadibrata, Thai Hau Koo, Raúl Alberto Jiménez-Castillo, Ronnie Fass

**Affiliations:** 1 Department of Medicine, MetroHealth Medical Center, Case Western Reserve University, Cleveland, OH USA; 2 Division of Gastroenterology and Hepatology, The Esophageal and Swallowing Center, MetroHealth Medical Center, Case Western Reserve University, Cleveland, OH USA

**Keywords:** Potassium-competitive acid blocker, Proton pump inhibitor, Histamine-2 receptor antagonist, Erosive esophagitis, Network meta-analysis

## Abstract

Randomized controlled trials (RCTs) suggest that potassium-competitive acid blockers (PCABs) are non-inferior to standard-dose proton pump inhibitors (PPIs) for erosive esophagitis (EE). However, direct comparative data against double-dose or dual-delayed release PPIs remain limited. This network meta-analysis (NMA) ranks the efficacy of PCABs and PPIs (standard, double, and dual-delayed-release doses) for EE healing and maintenance. A systematic search of MEDLINE, EMBASE, and the Cochrane Library from inception through February 2, 2025 (updated on December 15, 2025) identified RCTs comparing PCABs or PPIs in adults with endoscopically confirmed EE or previously healed EE. Primary outcomes were EE healing at weeks 4 and 8, and maintenance at 6 months. A frequentist NMA estimated risk ratios (RRs) with 95% confidence intervals (95%CI), using vonoprazan 20 mg once daily (QD) and 10 mg QD as reference for healing and maintenance studies, respectively; ranking metrics (P-scores) were used descriptively. A total of 99 RCTs were included. At week 4, several regimens showed similar healing rate to vonoprazan 20 mg QD, including linaprazan 75 mg BID, linaprazan 50 mg BID, and zastaprazan 20 mg QD. At week 8, zastaprazan 20 mg QD was significantly better than vonoprazan 20 mg QD (RR 1.10; 95%CI 1.01–1.21; P-score = 0.92). For maintenance at 6 months, several standard-dose regimens (including esomeprazole 40 mg QD and vonoprazan 20 mg QD) showed similar efficacy, whereas lower-dose PPIs performed worse. Adverse events were comparable across therapies. Zastaprazan 20 mg QD and esomeprazole 40 mg QD demonstrated the highest efficacy for EE healing and maintenance, respectively. Overall, PCABs did not consistently outperform dual-delayed release or double-dose PPIs.

**Supplementary Information:**

The online version contains supplementary material available at https://doi.org/10.1007/s10388-026-01222-2.

## Introduction

Proton pump inhibitors (PPIs) have been the cornerstone therapy for erosive esophagitis (EE) since the 1990s, largely replacing histamine-2 receptor antagonists (H2RAs) as first-line treatment [1, 2]. By providing more potent and sustained gastric acid suppression, PPIs achieve significantly higher mucosal healing rates across all grades of EE compared to H2RAs and, importantly, prevent the development of peptic strictures [1, 3]. Nevertheless, despite the utilization of PPIs over the last 35 years, there are still many areas of unmet needs [4]. It often takes several days of daily dosing to achieve maximal effect, nighttime acid control remains suboptimal, and healing of severe EE lesions (Los Angeles (LA) grade C/D) is less robust compared to mild to moderate disease [4–6]. Patients with LA grade C/D EE demonstrate lower healing rates with PPIs and frequently represent a significant portion of refractory gastroesophageal reflux disease (GERD) cases, underscoring the necessity for more aggressive acid suppression in advanced EE [6]. Consequently, the long-term management of EE frequently requires continuous maintenance therapy with PPIs to prevent disease recurrence.

In clinical practice, physicians commonly escalate therapy to double-dose PPIs, or advanced PPI formulations (e.g., dual-release PPIs such as dexlansoprazole) for difficult-to-treat EE or refractory reflux symptoms. Although standard once-daily PPIs provide sufficient relief for many EE patients, partial responders often benefit from twice-daily dosing [7, 8]. Indeed, doubling the PPI dose can improve esophageal healing rates in severe EE and mitigate nighttime reflux [9, 10]. The newer dexlansoprazole utilizes a dual delayed-release mechanism to extend acid suppression throughout the 24 hour. Dexlansoprazole 60 mg has demonstrated prolonged gastric pH control and flexible dosing times, resulting in high EE healing and maintenance rates [11]. Likewise, esomeprazole 40 mg, considered one of the most potent PPIs, has shown superior healing outcomes in severe EE compared to other PPI regimens in comparative studies [12]. These real-world therapeutic strategies, including double-dose (e.g., twice-daily) and dual-delayed-release PPI formulations, underscore the need for potent acid suppression beyond the capabilities of standard once-daily PPI dosing in EE patients with severe disease.

Potassium-competitive acid blockers (PCABs) represent a newer class of acid-suppressive medications, providing pharmacological advantages over PPIs [13]. PCABs, such as vonoprazan, reversibly inhibit the gastric H^+^K^+^-ATPase by potassium-competitive ionic binding, leading to rapid and potent acid suppression, independent of meal timing [5]. Unlike PPIs, which require activation in an acidic environment and pre-meal dosing, PCABs act directly on active and possibly on dormant proton pumps [14]. The pharmacodynamic and pharmacokinetic attributes of PCABs may provide therapeutic solutions to many areas of unmet need in GERD. Initial trials of vonoprazan in Asia have demonstrated at least comparable efficacy to lansoprazole for EE healing [6, 15]. More recently, the first global Phase 3 trial (PHALCON-EE) confirmed that vonoprazan 20 mg once daily is non-inferior to lansoprazole 30 mg once daily for 8-week healing, with notably faster early healing rates across all grades of EE, particularly those with LA grade C/D EE at week 2 [16]. However, most PCAB trials to date have compared against once-daily PPI dosing, whereas many GERD patients in clinical practice receive twice-daily or double-dose PPIs. Thus, it remains unclear whether PCABs offer incremental benefits over optimal PPI therapy, given the lack of head-to-head trials against more potent PPIs such as esomeprazole, double-dose, or the dual-delayed-release PPI.

Previous meta-analyses have concentrated on limited subsets, such as vonoprazan versus standard-dose PPIs, or included exclusively severe EE cases [17, 18] and current guidelines rank PPIs broadly above H2RAs without distinguishing between individual agents or dosing strategies. A critical knowledge gap persists regarding how the full spectrum of therapies, including standard-dose PPIs, double-dose PPIs, dual-delayed-release PPIs, and PCABs, compares in efficacy during both the healing and maintenance phases of EE. Therefore, we conducted a systematic review and network meta-analysis (NMA) to comprehensively evaluate and rank the relative efficacy of major acid-suppressive treatments for (i) the healing of EE and (ii) the maintenance of EE healing. Our aim was to determine whether PCABs provide additional benefit over conventional and higher doses of PPI therapy by evaluating relative efficacy and their statistical significance, rather than relying on rankings alone.

## Methods

The protocol for this NMA was registered with PROSPERO (CRD42025645182). This systematic review and NMA were conducted following the Preferred Reporting Items for Systematic Reviews and Meta-Analyses (PRISMA) guidelines and its extension for NMA (Supplementary File 1).

### Search strategy and selection criteria

We conducted a systematic literature search across three databases, including Ovid EMBASE, Ovid MEDLINE, and the Cochrane Library, from their inception through February 2, 2025, with an update performed on December 15, 2025. Only randomized controlled trials (RCTs) were considered eligible for study inclusion. For healing-phase studies, we included any RCT comparing at least two acid-suppressive therapies (PPI and/or PCAB) in adult patients with endoscopically confirmed EE, regardless of the severity criteria used. For maintenance-phase studies, we included RCTs comparing at least two acid-suppressive therapies (PPI and/or PCAB) or placebo in adults with endoscopically confirmed healed EE. Both double-blind and open-label RCTs were eligible. Quasi-randomized studies, defined as trials that used nonrandom allocation methods (e.g., alternation, date of birth, medical record number, or admission order) rather than a truly random sequence, were excluded due to known risk of bias and considered nonrandomized studies of intervention effects [19]. In cases of multiple publications reporting overlapping patient populations, the most comprehensive or the latest report was included. Reviews, letters to the editor, and conference abstracts were excluded.

Key search terms included “erosive esophagitis”, “reflux esophagitis”, “peptic esophagitis”, “gastroesophageal reflux disease”, or GERD; “proton pump inhibitor”, PPI, lansoprazole, omeprazole, esomeprazole, dexlansoprazole, rabeprazole, or pantoprazole; and “potassium competitive acid blocker”, PCAB, vonoprazan, tegoprazan, fexuprazan, revaprazan, keverprazan, or zastaprazan, captured both as MeSH terms and free text (Supplementary File 2). The search was unrestricted by language or publication date, and references from relevant articles were manually searched to identify additional studies.

Studies identified through database searches and manual handsearching were initially imported into EndNote 20 to remove duplicates and subsequently imported into Covidence for screening. After duplicate removal, two reviewers (DMS and THK) independently screened titles and abstracts, followed by full-text articles to assess eligibility. Full-text articles of potentially eligible studies were evaluated against the eligibility criteria, with disagreements resolved by consensus involving a third investigator (RJC).

### Outcomes measures

Primary outcomes were predefined as EE healing rates at weeks 4 and 8 for the healing phase, and maintenance or non-recurrence rates of healed EE at 6 months for the maintenance phase. Each study’s own definitions of “healed” (typically absence of mucosal breaks on endoscopy) and “relapse” (endoscopic recurrence of any EE break or study-defined treatment failure) were utilized. Secondary outcomes included healing rates at weeks 2, maintenance rates at 3 and 12 months, and the incidence of any adverse events (AEs) and serious AEs (SAEs) during both phases. In addition, EE severity was further categorized based on LA classification: (i) LA grade A/B equivalent (mild-to-moderate), (ii) LA grade C/D equivalent (severe), and (iii) LA grade B-D equivalent (conclusive GERD according to Lyon consensus 2.0). Given variations in EE staging methods in earlier acid suppressive therapy studies (e.g., Savary-Miller [SM], Hetzel-Dent [HD], and others), SM and HD grade I-II were considered equivalent with LA grade A/B, SM and HD grade III-IV with LA grade C/D, and SM and HD grade II-IV as equivalent with LA grade B-D.

### Data extraction

Two investigators (DMS and THK) independently extracted relevant data from each included study using a standardized form in a prespecified Microsoft Excel spreadsheet. The extracted data were compared, and discrepancies were resolved through discussion with a third investigator (RJC). Key information collected included sample size, study characteristics (authors, publication year, trial registration identifiers, study locations, single-center/multicenter), baseline patient characteristics (age, sex ratio, Helicobacter pylori status, EE severity grading at baseline when available), intervention details (drug name and dosage regimen), and outcomes of interest as described above. Only the intention-to-treat population was included, preserving original randomization integrity; thus, all dropouts were considered treatment failures.

### Quality assessment and risk of bias

Risk of bias for each RCT was independently assessed by two reviewers (DMS and THK) using the Cochrane Risk of Bias tool (RoB 2.0) [20], with discrepancies resolved through discussion with a third reviewer (RJC) until consensus was achieved. Five domains were evaluated: randomization process, deviations from intended interventions, missing outcome data, measurement of outcomes, and selection of reported results. Each domain was graded as “low”, “some concerns”, or “high” risk of bias [21]. Overall study risk of bias was categorized as follows: (i) “high” risk of bias if at least one domain indicated “high” risk; (ii) “some concerns” if at least one domain had “some concerns” but none had “high” risk of bias; and (iii) “low” risk if all domains had “low” risk of bias.

### Data synthesis and statistical analysis

Statistical analyses were performed using the “netmeta” package in RStudio (Vienna, Austria). Separate NMAs for healing and maintenance (recurrence prevention) phases were conducted using a frequentist framework for mixed treatment comparisons, anchored by standard references: vonoprazan 20 mg once daily for the healing phase and vonoprazan 10 mg once daily for maintenance. Treatment effects were expressed as pooled risk ratios (RRs) with 95% confidence intervals (95%CI) using the random effects models, consistent with standard meta-analysis practices [22]. Absolute risk differences were not separately reported, as our network meta-analysis framework focuses on relative effects. Treatment rankings were derived from P-scores for each intervention in the network, a frequentist ranking metric (analogous to the surface under the cumulative ranking curve [SUCRA]) based on the estimated relative effects and their uncertainty, representing the probability that a treatment is superior to others. P-scores do not represent the probability that a treatment is “truly superior,” and they should not be interpreted as probabilistic certainty, particularly when effect estimates are imprecise or when event counts are sparse. Rather, they should be interpreted as a descriptive ordering that must be considered alongside effect sizes (RRs and 95%CIs), particularly when differences are small or imprecise. Accordingly, we prioritized the magnitude and precision of relative effects (RRs with 95% CIs), using P-scores only as a descriptive summary of ranking. When 95%CIs overlapped by 1 for a comparison with the reference, we interpreted the regimens as not statistically different for that endpoint/timepoint. Multiple subgroup analyses were performed to determine if the ranking or magnitude of efficacy changes in different patient populations. We conducted analyses for LA grade A/B equivalents, LA grade C/D equivalents, and LA grade B-D equivalents. When ≥ 10 studies were available, publication bias and small-study effects were evaluated using comparison-adjusted funnel plots and Egger’s regression test, with p-value < 0.05 indicating significant small-study effects.

## Results

### Study selection

**Fig. 1:**
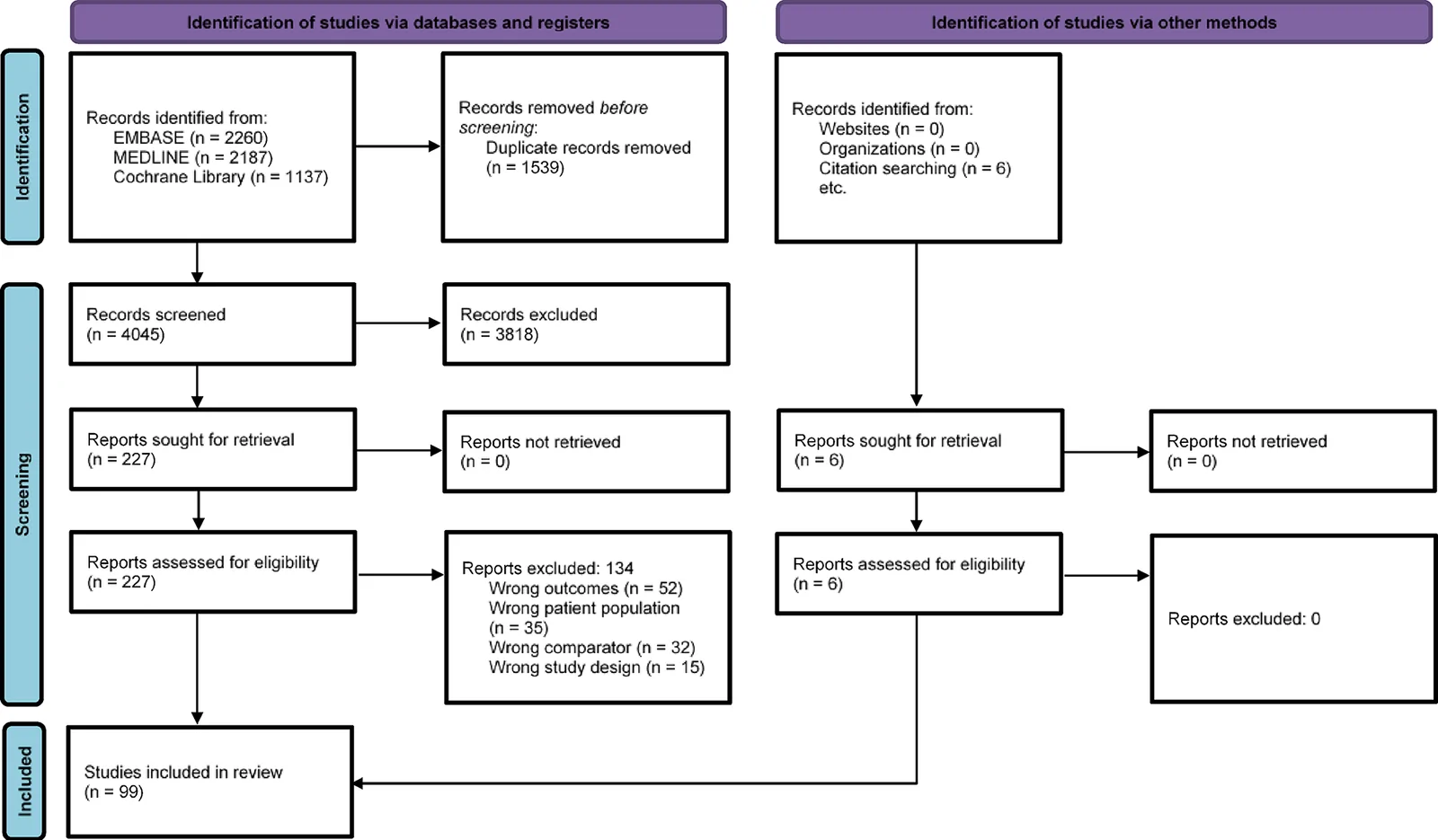
PRISMA flow diagram for study selection

The systematic search initially identified 5,584 citations from Ovid EMBASE, Ovid MEDLINE, and the Cochrane Library. After removing 1,539 duplicates, 4,045 records underwent title and abstract screening, resulting in the exclusion of 3,818 records. Of the remaining 227 full-text articles assessed for eligibility, 134 were excluded due to inappropriate outcomes (*n* = 52), ineligible patient populations (*n* = 35), inappropriate comparators (*n* = 32), or unsuitable study design, including quasi-randomized designs (*n* = 15). Additionally, six RCTs were identified via manual searches of reference lists. Ultimately, 99 RCTs met the inclusion criteria and were included in this NMA (Fig. 1).

### Study characteristics and risk of bias

Of the 99 included RCTs, 62 assessed the healing phase (Supplementary Files 3 and 4), 31 evaluated the maintenance phase (Supplementary Files 5 and 6), and six studies assessed both phases. The healing phase comprised 42 distinct treatment arms, while the maintenance phase had 22 treatment arms. Most studies were multicenter and predominantly conducted in Western populations. Baseline characteristics for healing studies and maintenance studies are detailed in Supplementary Files 3 and 5, respectively. PCAB regimens included vonoprazan, tegoprazan, linaprazan, fexuprazan, and zastaprazan.

Overall, the risk of bias was low to moderate for both healing and maintenance phases (Supplementary Files 7 and 8). Specifically, 4% (*n* = 3) of healing studies had a high risk of bias, primarily due to selective reporting of results, while 22% (*n* = 15) had some concerns, mostly related to missing outcome data and selective reporting of results (Supplementary File 7). For maintenance studies, 14% (*n* = 5) had a high risk of bias, mainly from missing outcome data, and 32% (*n* = 12) had some concerns (Supplementary File 8).

### Healing phase

#### Healing at weeks 4 and 8

**Fig. 2:**
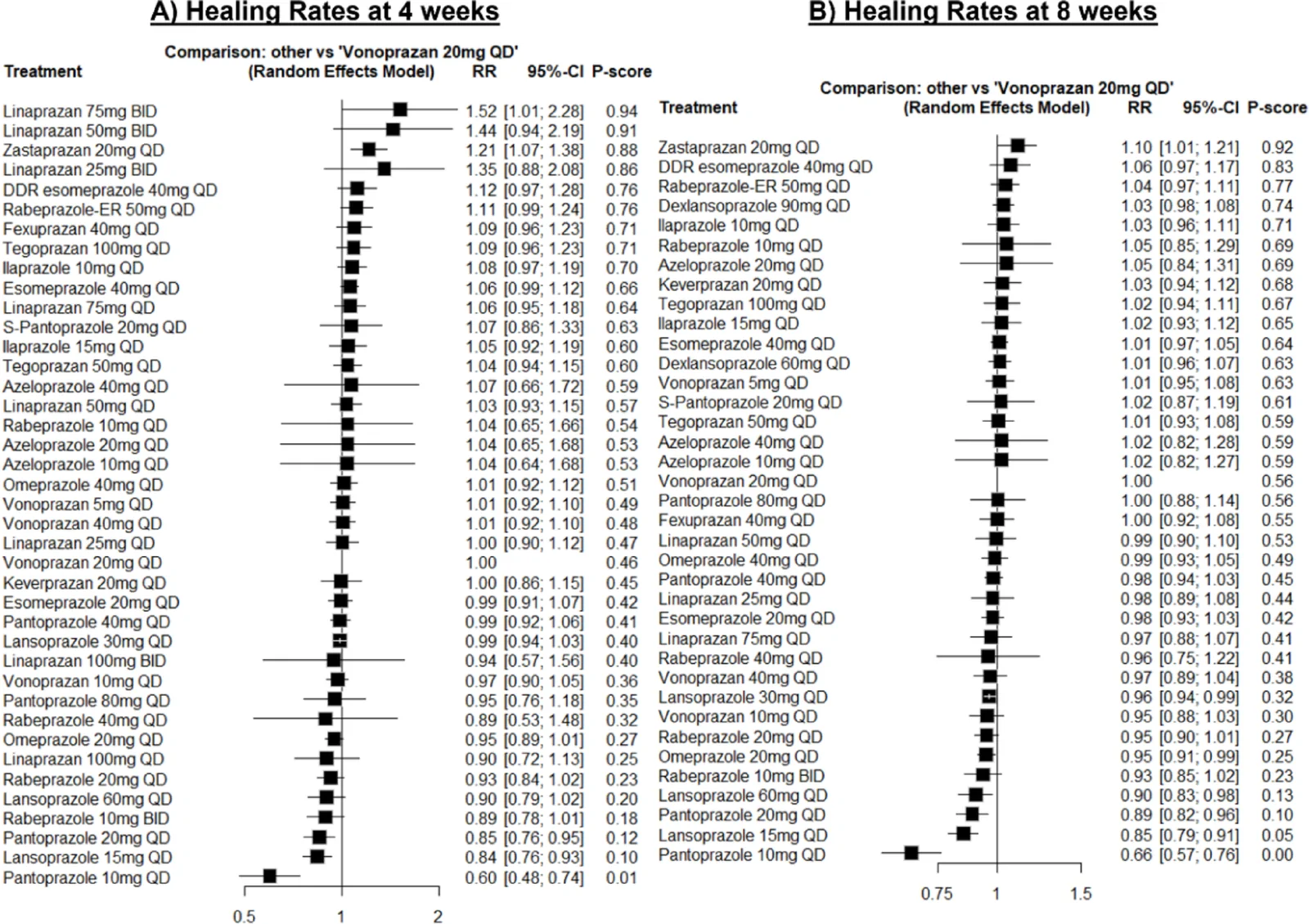
Network meta-analysis illustrating the healing rates of potassium-competitive acid blockers (PCABs) and proton pump inhibitors (PPIs) at **A** 4 weeks and **B** 8 weeks. All treatments are compared against vonoprazan 20 mg once daily (QD) as the reference standard and ranked according to their P-score. Treatments are ordered by P-score; higher P-scores indicate a more favorable ranking based on the estimated RRs, and should be interpreted alongside the effect estimates and 95%CIs

At week 4, Linaprazan 75 mg twice daily (BID; RR 1.52; 95%CI 1.01–2.28; P-score = 0.94), Linaprazan 50 mg BID (RR 1.44; 95%CI 0.94–2.19; P-score = 0.91), and Zastaprazan 20 mg once daily (QD; P-score = 0.88) were the three most efficacious therapies for EE healing compared with all other therapies. Notably, Zastaprazan 20 mg QD was significantly superior to Vonoprazan 20 mg QD (RR 1.21; 95%CI 1.07–1.38). Generally, PCABs ranked higher than PPIs (Fig. 2A). DDR esomeprazole 40 mg QD (RR 1.12; 95%CI 0.97–1.28; P-score = 0.76), Rabeprazole-ER 50 mg QD (RR 1.11; 95%CI 0.99–1.24; P-score = 0.76), Ilaprazole 10 mg QD (1.08; 95%CI 0.97–1.19; P-score = 0.70), Esomeprazole 40 mg QD (RR 1.06; 95%CI 0.99–1.12; P-score = 0.66), and S-Pantoprazole 20 mg QD (RR 1.07; 95%CI 0.86–1.33; P-score = 0.63) were the top five PPIs, exhibiting comparable efficacy to Vonoprazan 20 mg QD. Funnel plot analysis showed no significant small-study effects (*p* = 0.910; Supplementary File 9). The league table showing direct and indirect comparisons between treatment arms for EE healing at week 4 is shown in Supplementary File 10.

At week 8, Zastaprazan 20 mg QD (RR 1.10; 95%CI 1.01–1.21; P-score = 0.92), DDR esomeprazole 40 mg QD (RR 1.06; 95%CI 0.97–1.17; P-score = 0.83), and Rabeprazole-ER 50 mg QD (RR 1.04; 95%CI 0.97–1.11; P-score = 0.77) were most effective for healing of EE although showed similar efficacy with Vonoprazan 20 mg QD. PCABs and dual-delayed-release, double-dose, or more potent PPIs demonstrated similar RR compared with Vonoprazan 20 mg QD, whereas low-dose PPIs consistently showed significantly poorer 8-week healing rates (Fig. 2B). No small-study effects were detected for healing of EE at week 8 (*p* = 0.229; Supplementary File 11). The league table showing direct and indirect comparisons between treatment arms for EE healing at week 8 is shown in Supplementary File 12.

#### Healing at week 2

Early mucosal healing at week 2 (*n* = 9 treatment arms) identified Vonoprazan 20 mg QD as the most efficacious therapy (P-score = 0.87), followed by Vonoprazan 40 mg QD (RR 0.98; 95%CI 0.90–1.06; P-score = 0.73), Vonoprazan 10 mg QD (RR 0.96; 95%CI 0.88–1.04; P-score = 0.64), and Lansoprazole 60 mg QD (RR 0.96; 0.60–1.55; P-score = 0.57; Supplementary File 13). Lansoprazole 30 mg QD (RR 0.92; 95%CI 0.88–0.96; P-score = 0.41), Omeprazole 20 mg QD (RR 0.88; 95%CI 0.78–0.98; P-score = 0.26), and Lansoprazole 15 mg QD (RR 0.79; 95%CI 0.68–0.92; P-score = 0.05) showed significantly lower healing rates compared to Vonoprazan 20 mg QD. No significant small-study effects were observed (Egger’s p-value = 0.921; Supplementary File 14). The league table showing direct and indirect comparisons between treatment arms for healing of EE at week 2 is shown in Supplementary File 15.

#### Subgroup analysis by EE severity (based on LA grade A/B, C/D and B-D equivalents)

Among the severity-based subgroup analyses, differences were observed among patients with LA grade C/D-equivalent EE, in whom lower-dose or lower-potency PPI regimens generally performed less favorably than PCAB or optimized PPI regimens.

Based on analysis restricting to only EE patients with LA grade A/B equivalent, no treatment was significantly superior to Vonoprazan 20 mg QD. Lansoprazole 30 mg QD (RR 0.99; 95%CI 0.95–1.04; P-score = 0.32) and Vonoprazan 5 mg QD (RR 0.98; 95%CI 0.91–1.07; P-score = 0.25) ranked lowest at week 2 (Supplementary File 16 A). Fexuprazan 40 mg QD (RR 1.19; 95%CI 1.00-1.43; P-score = 0.92), Zastaprazan 20 mg QD (RR 1.13; 95%CI 0.99–1.30; P-score = 0.87), and Tegoprazan 50 mg QD (RR 1.12; 95%CI 1.00-1.26; P-score = 0.84) demonstrated the highest healing rates at week 4 despite similar efficacy between Vonoprazan, whereas only Pantoprazole 10 mg QD (RR 0.67; 95%CI 0.54–0.85; P-score = 0.02) was significantly inferior to Vonoprazan 20 mg QD (Supplementary File 16B). At week 8, all interventions were similar in efficacy to Vonoprazan 20 mg QD, except for significantly lower efficacy noted with Pantoprazole 20 mg QD (RR 0.93; 95%CI 0.86-1.00; P-score = 0.11) and 10 mg QD (RR 0.77; 95%CI 0.67–0.90; P-score = 0.00) at 8 weeks (Supplementary File 16 C).

For LA grade C/D equivalent patients, Vonoprazan 5 mg QD (RR 0.80; 95%CI 0.69–0.93; P-score = 0.15) and Lansoprazole 30 mg QD (RR 0.80; 95%CI 0.73–0.87; P-score = 0.11) showed significantly lower efficacy at 2 weeks, while the remainder had similar healing rates (Supplementary File 17 A). Linaprazan 75 mg BID (RR 2.20; 95%CI 0.96–5.04; P-score = 0.97), Linaprazan 50 mg BID (RR 1.89; 95%CI 0.78–4.56; P-score = 0.92), and Zastaprazan 20 mg QD (RR 1.15; 95%CI 0.94–1.40; P-score = 0.85) ranked highest at week 4, while Rabeprazole-ER 50 mg QD (RR 1.02; 95%CI 0.89–1.17; P-score = 0.76), Esomeprazole 40 mg QD (RR 0.97; 95%CI 0.88–1.08; P-score = 0.67), and DDR esomeprazole 40 mg QD (RR 1.03; 95%CI 0.67–1.60; P-score = 0.67) were the three highest performing PPIs with similar healing rates as Vonoprazan 20 mg QD (Supplementary File 17B). At Week 8, Zastaprazan 20 mg QD (RR 1.16; 95%CI 0.73–1.84; P-score = 0.82), Rabeprazole-ER 50 mg QD (RR 1.01; 95%CI 0.88–1.16; P-score = 0.77), and Vonoprazan 20 mg QD (P-score = 0.77) were the three highest performing medications although with statistically similar healing rates (Supplementary File 17 C). Lansoprazole 15 mg QD (RR 0.61; 95%CI 0.48–0.78) and 30 mg QD (RR 0.89; 95%CI 0.82–0.96; P-score = 0.38), Omeprazole 20 mg QD (RR 0.86; 95%CI 0.77–0.96; P-score = 0.29) and 40 mg QD (RR 0.84; 95%CI 0.72–0.98; P-score = 0.27), and Pantoprazole 10 mg QD (RR 0.42; 95%CI 0.29–0.61; P-score = 0.00) and 20 mg QD (RR 0.77; 95%CI 0.60–0.99; P-score = 0.19) had significantly lower healing rates than Vonoprazan 20 mg QD at 8 weeks.

Among LA grade B-D equivalent EE patients, Vonoprazan 5 mg QD (RR 0.80; 95%CI 0.69–0.93; P-score = 0.36), Lansoprazole 30 mg QD (RR 0.80; 95%CI 0.73–0.87; P-score = 0.35), Omeprazole 20 mg QD (RR 0.76; 95%CI 0.66–0.87; P-score = 0.21), and Lansoprazole 15 mg QD (RR 0.68; 95%CI 0.58–0.81; P-score = 0.03) had significantly inferior healing rates at Week 2 (Supplementary File 18 A). At week 4, Linaprazan 75 mg BID (RR 2.20; 95%CI 0.96–5.04; P-score = 0.97), Linaprazan 50 mg BID (RR 1.89; 95%CI 0.78–4.56; P-score = 0.92), and Zastaprazan 20 mg QD (RR 1.15; 95%CI 0.94–1.40; P-score = 0.85) were the three highest performing medications despite none performed significantly better than Vonoprazan 20 mg QD (Supplementary File 18B). In comparison, at Week 8, no intervention performed significantly better than Vonoprazan 20 mg QD, meanwhile lower-dose or lower-potency PPIs had significantly worse healing rates than Vonoprazan 20 mg QD (Supplementary File 18 C).

#### Any and serious adverse events

**Fig. 3:**
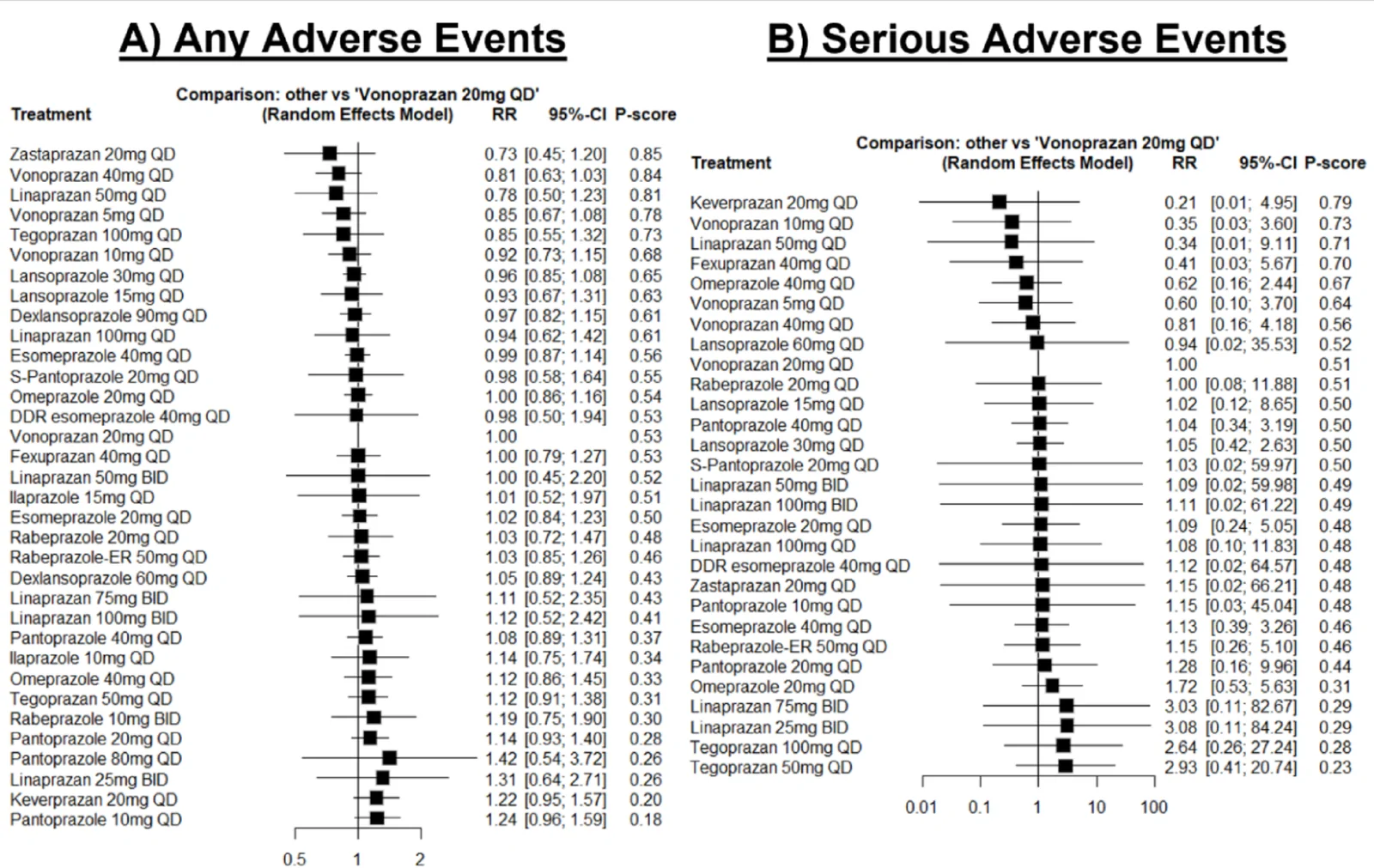
Network meta-analysis for **A** any adverse events and **B** serious adverse events of potassium-competitive acid blockers (PCABs) and proton pump inhibitors (PPIs) during treatment for healing erosive esophagitis. Vonoprazan 20 mg once daily (QD) serves as the reference standard. Treatments are ordered by P-score; higher P-scores indicate a more favorable ranking (lower estimated RR for events)

Overall, AEs and SAEs were similar across all treatment arms compared with Vonoprazan 20 mg QD (Fig. 3A and B). There were no significant small-study effects for any AEs (Supplementary File 19) and for SAEs (Supplementary File 20).

### Maintenance phase

#### Maintenance rates at 6 months

**Fig. 4:**
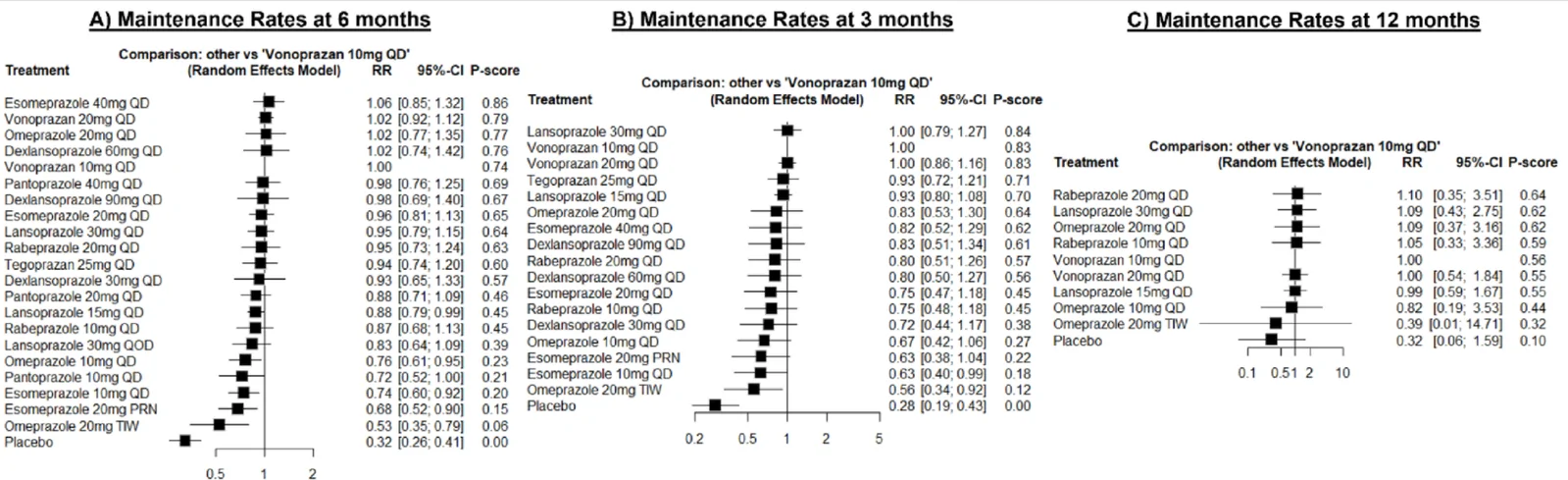
Network meta-analysis of maintenance of healing rates of potassium-competitive acid blockers (PCABs) and proton pump inhibitors (PPIs) at **A** 6 months, **B** 3 months, and **C** 12 months. Vonoprazan 10 mg once daily (QD) is the reference treatment. Treatments are ordered by P-score; higher P-scores indicate a more favorable ranking based on estimated RRs, interpreted alongside effect sizes and 95%CIs

At six months, Esomeprazole 40 mg QD (RR 1.06; 95%CI 0.85–1.32; P-score = 0.86), Vonoprazan 20 mg QD (RR 1.02; 95%CI 0.92–1.12; P-score = 0.79), and Omeprazole 20 mg QD (RR 1.02; 95%CI 0.77–1.35; P-score = 0.77) demonstrated the highest maintenance rates of healed EE across all medications, although no interventions were statistically significantly better than Vonoprazan 10 mg QD as reference group (Fig. 4A). Lansoprazole 15 mg QD (RR 0.88; 95%CI 0.79–0.99; P-score = 0.45), Omeprazole 10 mg QD (RR 0.76; 95%CI 0.61–0.95; P-score = 0.23), Esomeprazole 10 mg QD (RR 0.74; 95%CI 0.60–0.92; P-score = 0.20), Esomeprazole 20 mg as needed (PRN; RR 0.68; 95%CI 0.52–0.90; P-score = 0.15), and Omeprazole 20 mg three times weekly (TIW; RR 0.53; 95%CI 0.35–0.79; P-score = 0.06) had significantly lower maintenance rates compared to Vonoprazan 10 mg QD. The league table showing direct and indirect comparisons between treatment arms for maintenance of healed EE at 6 months is shown in Supplementary File 21. Comparison-adjusted funnel plot showed no significant small-study effect for maintenance of EE healing at 6 months (Egger’s test p-value = 0.502; Supplementary File 22). Subgroup analysis based on LA Grade A/B, C/D, and B-D equivalent showed that Lansoprazole 15 mg QD and Esomeprazole 20 mg PRN had consistently significantly lower maintenance of healed EE (Supplementary File 23). In addition, for patients with baseline LA grade B-D, Rabeprazole 10 mg QD (RR 0.51; 95%CI 0.30–0.89; P-score = 0.20) and Omeprazole 10 mg QD (RR 0.38; 95%CI 0.24–0.60; P-score = 0.08) also performed significantly worse than Vonoprazan 10 mg QD in maintaining EE healing (Supplementary File 23 C).

#### Maintenance rates at 3 and 12 months

At 3 months (Fig. 4B), Esomeprazole 10 mg QD (RR 0.63; 95%CI 0.40–0.99; P-score = 0.18), Omeprazole 20 mg TIW (RR 0.56; 95%CI 0.34–0.92; P-score = 0.12), and placebo (RR 0.28; 95%CI 0.19–0.43; P-score = 0.00) performed statistically significantly worse than Vonoprazan 10 mg QD. The league table showing direct and indirect comparisons between treatment arms for maintenance of healed EE at 3 months is shown in Supplementary File 24. There were no statistically significant small-study effects for any adverse events (*p* = 0.657; Supplementary File 25). By 12 months (Fig. 4C), no statistically significant difference was noted between Vonoprazan 10 mg QD versus other treatments, although Omeprazole 20 mg TIW (RR 0.39; 95%CI 0.01–14.71; P-score = 0.32) and placebo (RR 0.32; 95%CI 0.06–1.59; P-score = 0.10) had the lowest maintenance of healed EE. The league table showing both direct and indirect comparison between each treatment arm for maintenance of healed EE at 12 months is shown in Supplementary File 26. There were no statistically significant small-study effects for maintenance of healed EE at 12 months (*p* = 0.957; Supplementary File 27).

#### Any and serious adverse events

**Fig. 5:**
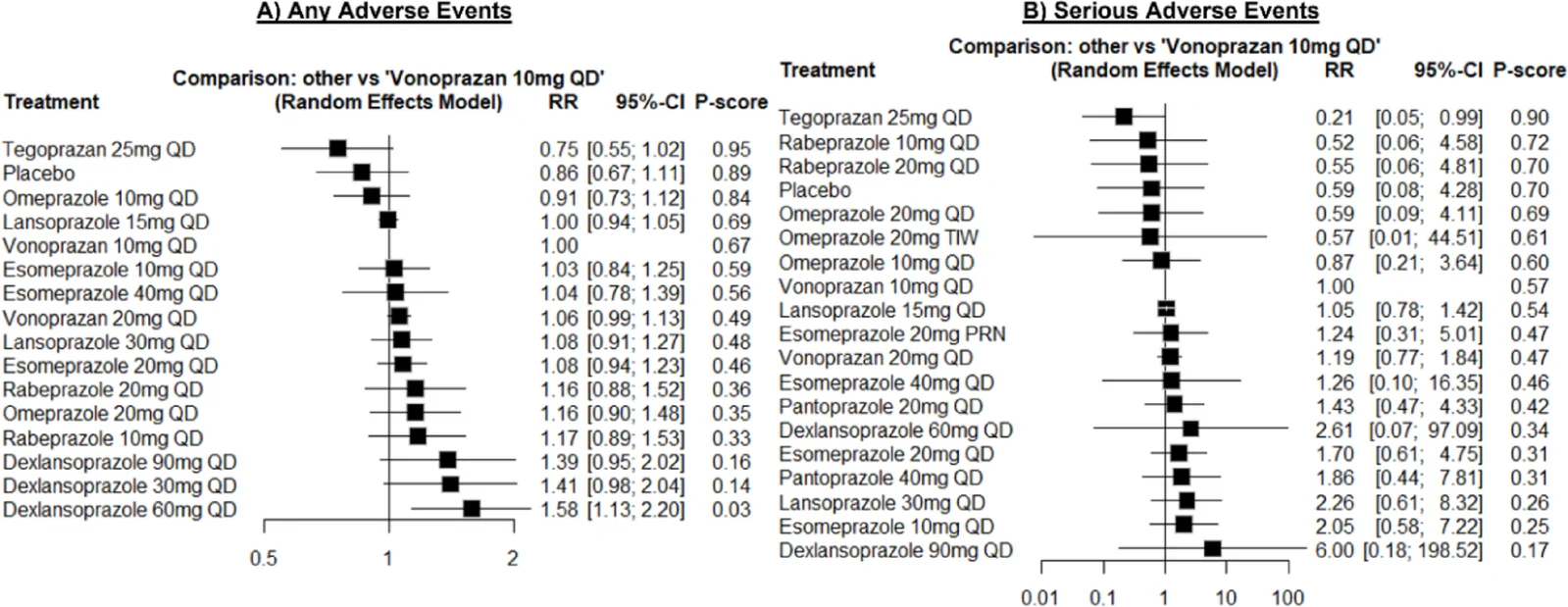
Network meta-analysis for **A** any adverse events and **B** serious adverse events of potassium-competitive acid blockers (PCABs) and proton pump inhibitors (PPIs) in studies assessing maintenance therapies for erosive esophagitis. Vonoprazan 10 mg once daily (QD) serves as the reference standard. Treatments are ordered by P-score; higher P-scores indicate a more favorable ranking (lower estimated RR for events), interpreted alongside absolute event risks and event counts

Maintenance regimens were generally well tolerated; however, Dexlansoprazole 60 mg QD showed significantly higher rates of any adverse events compared to Vonoprazan 10 mg QD (RR 1.58; 95%CI 1.13–2.20; P-score 0.03) (Fig. 5A). Funnel plot showed no significant small-study effects (Egger’s test *p* = 0.336; Supplementary File 28). For any serious adverse events, generally rates were similar across all treatment groups when compared to Vonoprazan 10 mg QD, with the exception that Tegoprazan 25 mg QD had statistically significantly lower rates of serious adverse events (RR 0.21; 95%CI 0.05–0.99; P-score 0.90) (Fig. 5B). No statistically significant small-study effect was noted (Egger’s test p-value = 0.409; Supplementary File 29).

## Discussion

This NMA provides a comprehensive comparison of all acid-suppressive therapies for EE, encompassing 99 RCTs (62 RCTs evaluated the healing phase, 31 RCTs evaluated the maintenance phase, and six RCTs evaluated both the healing and maintenance phases). We evaluated the healing phase and maintenance phases of EE at different time-points across various therapeutic options, including standard-dose PPI, escalated PPI dosing, and PCABs such as Vonoprazan, Tegoprazan, Linaprazan, Fexuprazan, and Zastaprazan. For EE healing, Zastaprazan 20 mg QD ranked highest at week 8 (P-score 0.92) and third at week 4, while Linaprazan 75 mg and 50 mg BID led at week 4. Maintenance therapy rankings favored Esomeprazole 40 mg QD, Vonoprazan 20 mg QD, Lansoprazole 30 mg QD and Rabeprazole 20 mg QD. Overall, while PCABs and escalated doses of PPI generally outperformed standard-dose PPI therapies, standard-dose PPIs still provide robust healing and maintenance of EE.

For EE healing, our results suggest that more intensive acid-suppressive regimens (across both PCAB and optimized PPI strategies) can be associated with slightly higher healing rates than standard-dose PPI regimens. PCAB regimens such as Zastaprazan 20 mg QD and Linaprazan (50–75 mg BID) ranked among higher-performing interventions at weeks 4–8 although non-inferior if not only marginally better than Vonoprazan 20 mg QD, aligning with their pharmacological advantage of a rapid onset and prolonged gastric acid control over PPIs [23]. These pharmacologic features may contribute to clinical efficacy [24, 25]. However, our NMA evaluates clinical outcomes and does not directly measure intragastric pH or the onset/duration of acid suppression; therefore, mechanistic inferences are presented cautiously. Importantly, our network found that only Zastaprazan 20 mg QD and Linaprazan 75 mg and 50 mg BID were significantly superior to Vonoprazan, indicating modest but meaningful incremental efficacy gains. However, the between-regimen differences among top-ranked options were generally small and often indirect and should be interpreted primarily through the RRs and their 95%CIs, rather than rank order.

Optimized PPI therapies also demonstrated nearly comparable healing outcomes to Vonoprazan 20 mg QD. Dual delayed-release Esomeprazole 40 mg, which is similar in pharmacokinetics to the currently marketed Dexlansoprazole, Rabeprazole-ER 50 mg and double-dose Dexlansoprazole 90 mg ranked closely behind top PCABs (P-scores 0.83, 0.77, and 0.74, respectively), confirming that aggressive dosing or advanced formulations significantly enhance PPI efficacy, approaching that of PCABs. Clinically, EE patients unresponsive to standard-dose PPI may benefit equally from either switching to a PCAB or escalating PPI therapy (e.g., BID or dual-delayed release formulations). Previous studies demonstrated that doubling the PPI dose improves healing, particularly in severe (LA grade C/D) EE, where standard-dose regimens fail in up to 35% of patients [26]. Our analysis further clarifies that no single strategy universally outperforms others; for instance, Vonoprazan 20 mg QD, although highly effective, showed comparable efficacy to Dexlansoprazole and dual-delayed release Esomeprazole, and was surpassed by Zastaprazan and Linaprazan BID. These findings challenge a simplistic assumption that the PCAB drug category uniformly outperforms PPIs. Rather, our network estimates are consistent with the broader concept that more intensive acid-suppressive regimens (via higher dose and/or formulation) tend to yield higher healing rates. However, because this NMA compares clinical regimens (drug, dose, and formulation) rather than directly measuring intragastric pH, we cannot attribute observed differences solely to “acid suppression” or infer a class-independent mechanism. Accordingly, we interpret the results as evidence of comparative effectiveness across regimens, not as proof that “optimal acid suppression” is the sole determinant.

Disease severity emerged as another significant factor. Our broad EE population analysis aligns with subgroup analyses reported in the literature, showing that PCAB advantages are more pronounced in severe EE. A recent meta-analysis of Grade C/D EE found that Vonoprazan 20 mg provided the highest likelihood of healing (SUCRA ~ 0.89) [17], significantly outperforming PPIs in severe EE but not in mild cases [27]. This aligns with our findings, as most trials enrolled mixed or predominantly low-grade EE (e.g., the Zastaprazan vs. Esomeprazole trial was mostly LA Grade A/B patients) [23]. Therefore, in mild EE cases, the healing rates with any potent acid suppression (PPI or PCAB) approach 100%, minimizing between-drug differences. In contrast, for patients with severe EE, PCABs hold a more meaningful advantage, as they can heal lesions that might be otherwise persistent on PPIs [27]. Clinically, this supports a tailored approach: using PCABs or double-dose PPIs for severe or refractory EE, while standard-dose PPIs remain adequate for most mild-to-moderate cases.

In the maintenance phase, aggressive acid suppression was crucial for preventing EE recurrence. The highest-ranked regimens were standard-dose PPIs (Esomeprazole 40 mg, Lansoprazole 30 mg, and Rabeprazole 20 mg), which are considered double to the recommended half-dose PPI for preventing EE recurrence. This suggests that many patients may require standard-dose PPI therapy at least in the short to medium term to remain in remission. A key question is whether PCABs offer superior long-term protection against EE relapse compared with PPIs. Our NMA suggests that PCABs are not significantly more effective for maintenance treatment compared to standard-dose PPI. Vonoprazan 20 mg QD, for instance, ranked among the best therapies at the 3-month mark (comparable to Lansoprazole 30 mg QD in terms of efficacy). However, by 6 months, the highest ranks were held by Esomeprazole 40 mg QD, Vonoprazan 20 mg QD, and Omeprazole 20 mg QD. In the pivotal trial by Laine et al., Von*o*prazan 20 mg and even 10 mg daily both achieved higher 24-week maintenance rates than Lansoprazole 15 mg daily, with an absolute benefit of 7–9% (and up to 15% higher maintenance specifically in patients with LA grades C/D) [16]. However, when PCAB is compared with higher doses of PPI, the difference in efficacy diminishes.

In our maintenance network, several standard-dose regimens achieved similar effectiveness for relapse prevention, whereas lower-dose, PRN, or intermittent schedule regimens were more likely to be inferior in the available trials. We did not detect a consistent efficacy advantage of PCAB regimens over standard-dose PPI regimens at 6 months, as reflected by overlapping 95%CIs for the key comparisons. Because tolerability profiles, patient preferences, access, and cost were not uniformly or comparably measured across trials, and because safety event rates were often low, this NMA does not directly establish that maintenance choices should be driven primarily by tolerability or convenience. Therefore, clinicians should be cautious about routine step-down to “half-dose” or intermittent therapy immediately after healing, particularly in patients at higher relapse risk (e.g., prior LA Grade C/D, recurrent disease), and instead individualize dose reduction based on response and recurrence history. When efficacy appears similar among daily regimens, real-world factors, including prior response, patient preference, availability/coverage, and cost, may reasonably guide shared decision-making. Altogether, our maintenance-phase findings reinforce the guideline recommendations that patients with healed EE, especially those with frequent relapses or complications, should remain on daily PPI therapy long-term [28].

This study has several notable strengths that lend credibility and clinical relevance to our findings. First, to our knowledge, this is the most comprehensive NMA to date, examining both the healing and maintenance phases of EE across 99 RCTs. Unlike prior analyses that assessed acute or maintenance treatment in isolation, this dual-phase view offers insights into how initial healing predicts long-term control. Second, we compared the widest range of therapies to date, from standard PPIs to double-dose regimens, dual-delayed release formulations, and new PCABs. Including newer agents, such as Linaprazan and Zastaprazan, ensures that our findings reflect the latest evidence. Third, the network meta-analytic design enabled comparisons that had not been examined in direct trials. We indirectly compared treatments lacking head-to-head trials, such as PCABs vs. double-dose PPI, aiding with real-world decisions such as switching vs. escalation. Additionally, our analysis used a rigorous methodology (a frequentist network framework with P-score ranking), with sensitivity analyses to confirm its robustness. Finally, the large sample size enhances the power and precision of our estimates, and the consistency of the findings with known clinical pharmacology (e.g., greater acid suppression yields better outcomes) supports the validity of our findings.

Despite its strengths, this study had several limitations. Heterogeneity across trials is a key factor in this regard. The included RCTs spanned several decades and included diverse geographic regions, with differences in patient populations and trial designs. For instance, some studies focused on patients with severe EE, while others included mostly mild cases, which could affect healing rates and, thereby, the relative efficacy observed for a given drug. We attempted to account for this by analyzing a large aggregate population, but unmeasured heterogeneity remains; for example, variations in Helicobacter pylori prevalence or NSAID use across regions might influence mucosal healing independent of the acid suppressant used. There is evidence that regional differences exist in the response to EE therapy, and one meta-analysis noted that Vonoprazan’s relative advantage over PPIs may differ between Western and Asian cohorts [27], potentially reflecting differences in case-mix (including EE severity) and/or background pharmacogenetics. PPI effectiveness can be influenced by CYP2C19 metabolizer status, which varies across populations; however, genotype was not consistently measured or reported across included trials. While many PCABs are less dependent on CYP2C19 than PPIs (with metabolism largely via other pathways), we did not have sufficient trial-level data to stratify the network by genotype, ancestry, or region, and between-trial differences in these factors could contribute to heterogeneity. Our network combined trials from Asia, Europe, and North America; while improving generalizability, it may also mask region/subgroup-specific outcomes. Accordingly, our comparative estimates and rankings should be interpreted as average effects across mixed populations, and future studies should evaluate whether relative treatment effects differ by region, EE severity (e.g., LA grades C/D), and/or CYP2C19 metabolizer status (e.g., via prespecified subgroup analyses or network meta-regression when data permit).

In conclusion, this NMA summarizes comparative effectiveness and safety of PCAB- and PPI-based regimens for EE healing and maintenance and affirms that potent acid suppression is the cornerstone of both EE healing and preventing its recurrence. PCABs, such as but not limited to Vonoprazan, Linaprazan, and Zastaprazan, offer effective alternatives, particularly for patients with partial PPI response or severe EE, achieving healing rates that slightly exceed or at least match optimized PPI therapy. However, PPIs remain highly effective, especially when escalated doses or advanced formulations are used, a strategy that, in many cases, can bridge the gap in PCAB-level efficacy. Both PCABs and PPIs were similarly effective for maintenance, supporting individualized therapy based on tolerance, adherence, and access. Practical considerations should also be weighed carefully when comparing regimens. Several PCAB regimens included in this network were administered twice daily, and therefore, dosing convenience should not be generalized to the entire PCAB class. However, unlike conventional PPIs, PCABs are not dependent on pre-meal administration, which may offer greater dosing flexibility in clinical practice. In addition, dexlansoprazole, a dual delayed-release formulation, can be given once daily, regardless of meals. Accordingly, when efficacy is similar across regimens, treatment selection may reasonably be individualized based on practical considerations such as dosing frequency, meal timing, adherence, availability, cost, and patient preference. Future research should focus on head-to-head trials of PCABs versus double-dose PPIs and long-term outcomes beyond one year. Ongoing pharmacovigilance is needed to ensure long-term safety as these therapies enter broader use.

## Supplementary Information

Below is the link to the electronic supplementary material.

Supplementary Material 1

Supplementary Material 2

Supplementary Material 3

Supplementary Material 4

Supplementary Material 5

Supplementary Material 6

Supplementary Material 7

Supplementary Material 8

Supplementary Material 9

## Data Availability

The data are available upon request from the first and corresponding authors.

## References

[1] Wang WH, Huang JQ, Zheng GF, Xia HH, Wong WM, Lam SK, et al. Head-to-head comparison of H2-receptor antagonists and proton pump inhibitors in the treatment of erosive esophagitis: a meta-analysis. World J Gastroenterol. 2005;11(26):4067–77. 10.3748/wjg.v11.i26.4067 PMC4502104 15996033

[2] Strand DS, Kim D, Peura DA. 25 Years of proton pump inhibitors: a comprehensive review. Gut Liver. 2017;11(1):27–37. 10.5009/gnl15502 PMC5221858 27840364

[3] Chiba N, De Gara CJ, Wilkinson JM, Hunt RH. Speed of healing and symptom relief in grade II to IV gastroesophageal reflux disease: a meta-analysis. Gastroenterology. 1997;112(6):1798–810. 10.1053/gast.1997.v112.pm9178669 9178669

[4] Yadlapati R, Hubscher E, Pelletier C, Jacob R, Brackley A, Shah S. Induction and maintenance of healing in erosive esophagitis in the United States. Expert Rev Gastroenterol Hepatol. 2022;16(10):967–80. 10.1080/17474124.2022.2134115 36254610

[5] Oshima T, Miwa H. Potent potassium-competitive acid blockers: a new era for the treatment of acid-related diseases. J Neurogastroenterol Motil. 2018;24(3):334–44. 10.5056/jnm18029 PMC6034668 29739175

[6] Ashida K, Sakurai Y, Nishimura A, Kudou K, Hiramatsu N, Umegaki E, et al. Randomised clinical trial: a dose-ranging study of vonoprazan, a novel potassium-competitive acid blocker, vs. lansoprazole for the treatment of erosive oesophagitis. Aliment Pharmacol Ther. 2015;42(6):685–95. 10.1111/apt.13331 PMC5014135 26201312

[7] Fass R. Healing erosive esophagitis with a proton pump inhibitor: the more the merrier? Am J Gastroenterol. 2012;107(4):531–3. 10.1038/ajg.2012.25 22475962

[8] Zhang H, Yang Z, Ni Z, Shi Y. A meta-analysis and systematic review of the efficacy of twice daily PPIs versus once daily for treatment of gastroesophageal reflux disease. Gastroenterol Res Pract. 2017;2017:9865963. 10.1155/2017/9865963 PMC5585660 28912807

[9] Yadlapati R, Gyawali CP, Pandolfino JE. AGA clinical practice update on the personalized approach to the evaluation and management of GERD: expert review. Clin Gastroenterol Hepatol. 2022;20(5):984–e941. 10.1016/j.cgh.2022.01.025 PMC9838103 35123084

[10] Peghini PL, Katz PO, Bracy NA, Castell DO. Nocturnal recovery of gastric acid secretion with twice-daily dosing of proton pump inhibitors. Am J Gastroenterol. 1998;93(5):763–7. 10.1111/j.1572-0241.1998.221_a.x 9625124

[11] Davies SL. Dexlansoprazole for the treatment of esophagitis and GERD. Drugs Today (Barc). 2010;46(2):75–80. 10.1358/dot.2010.46.2.1437714 20393635

[12] Edwards SJ, Lind T, Lundell L, Das R. Systematic review: standard- and double-dose proton pump inhibitors for the healing of severe erosive oesophagitis—a mixed treatment comparison of randomized controlled trials. Aliment Pharmacol Ther. 2009;30(6):547–56. 10.1111/j.1365-2036.2009.04077.x 19558609

[13] Wong N, Reddy A, Patel A. Potassium-competitive acid blockers: present and potential utility in the armamentarium for acid peptic disorders. Gastroenterol Hepatol (N Y). 2022;18(12):693–700. PMC9972603 36865588

[14] Mulford DJ, Leifke E, Hibberd M, Howden CW. The effect of food on the pharmacokinetics of the potassium-competitive acid blocker vonoprazan. Clin Pharmacol Drug Dev. 2022;11(2):278–84. 10.1002/cpdd.1009 PMC9291755 34431240

[15] Xiao Y, Zhang S, Dai N, Fei G, Goh KL, Chun HJ, et al. Phase III, randomised, double-blind, multicentre study to evaluate the efficacy and safety of vonoprazan compared with lansoprazole in Asian patients with erosive oesophagitis. Gut. 2020;69(2):224–30. 10.1136/gutjnl-2019-318365 PMC6984055 31409606

[16] Laine L, DeVault K, Katz P, Mitev S, Lowe J, Hunt B, et al. Vonoprazan versus lansoprazole for healing and maintenance of healing of erosive esophagitis: a randomized trial. Gastroenterology. 2023;164(1):61–71. 10.1053/j.gastro.2022.09.041 36228734

[17] Zhuang Q, Chen S, Zhou X, Jia X, Zhang M, Tan N, et al. Comparative efficacy of P-CAB vs proton pump inhibitors for grade C/D esophagitis: a systematic review and network meta-analysis. Am J Gastroenterol. 2024;119(5):803–13. 10.14309/ajg.0000000000002714 38345252

[18] Cheng Y, Liu J, Tan X, Dai Y, Xie C, Li X, et al. Direct comparison of the efficacy and safety of vonoprazan versus proton-pump inhibitors for gastroesophageal reflux disease: a systematic review and meta-analysis. Dig Dis Sci. 2021;66(1):19–28. 10.1007/s10620-020-06141-5 32095968

[19] Sharma Waddington H, Wilson DB, Pigott T, Stewart G, Aloe AM, Tugwell P, et al. Quasi-experiments are a valuable source of evidence about effects of interventions, programs and policies: commentary from the Campbell Collaboration Study Design and Bias Assessment Working Group. J Clin Epidemiol. 2022;152:311–3. 10.1016/j.jclinepi.2022.11.005 36356851

[20] Sterne JAC, Savović J, Page MJ, Elbers RG, Blencowe NS, Boutron I, et al. RoB 2: a revised tool for assessing risk of bias in randomised trials. BMJ. 2019;366:l4898. 10.1136/bmj.l4898 31462531

[21] Cumpston M, Li T, Page MJ, Chandler J, Welch VA, Higgins JP, et al. Updated guidance for trusted systematic reviews: a new edition of the cochrane handbook for systematic reviews of interventions. Cochrane Database Syst Rev. 2019;10(10):Ed000142. 10.1002/14651858.ED000142 PMC10284251 31643080

[22] Deeks JJ. Issues in the selection of a summary statistic for meta-analysis of clinical trials with binary outcomes. Stat Med. 2002;21(11):1575–600. 10.1002/sim.1188 12111921

[23] Oh JH, Kim HS, Cheung DY, Lee HL, Lee DH, Kim GH, et al. Randomized, double-blind, active-controlled phase 3 study to evaluate efficacy and safety of zastaprazan compared with esomeprazole in erosive esophagitis. Am J Gastroenterol. 2025;120(2):353–61. 10.14309/ajg.0000000000002929 38976448

[24] Zhang M, Xiao Y, Chen M. The role of vonoprazan in patients with erosive esophagitis. Th Adv Gastroenterol. 2022;15:17562848221122623. 10.1177/17562848221122623 PMC9478709 36117573

[25] Shin JM, Inatomi N, Munson K, Strugatsky D, Tokhtaeva E, Vagin O, et al. Characterization of a novel potassium-competitive acid blocker of the gastric H,K-ATPase, 1-[5-(2-fluorophenyl)-1-(pyridin-3-ylsulfonyl)-1H-pyrrol-3-yl]-N-methylmethanamine monofumarate (TAK-438). J Pharmacol Exp Ther. 2011;339(2):412–20. 10.1124/jpet.111.185314 PMC3199995 21828261

[26] Higuchi K, Joh T, Nakada K, Haruma K. Is proton pump inhibitor therapy for reflux esophagitis sufficient? a large real-world survey of Japanese patients. Intern Med. 2013;52(13):1447–54. 10.2169/internalmedicine.52.0349 23812190

[27] Simadibrata DM, Lesmana E, Fass R. Vonoprazan is superior to lansoprazole for healing of severe but not mild erosive esophagitis: a systematic review with meta-analysis of randomized controlled trials. J Gastroenterol Hepatol. 2024;39(6):988–99. 10.1111/jgh.16486 38353152

[28] Uemura N, Kinoshita Y, Haruma K, Kushima R, Yao T, Akiyama J, et al. Vonoprazan as a long-term maintenance treatment for erosive esophagitis: VISION, a 5-year, randomized, open-label study. Clin Gastroenterol Hepatol. 2025;23(5):748–e575. 10.1016/j.cgh.2024.08.004 39209187

