# Supplementary material for: Potassium-competitive acid blockers and proton-pump inhibitors for the healing and maintenance of erosive esophagitis: a comprehensive and updated systematic review with network meta-analysis
Source: Esophagus. 2026 Jun 15;23(4):702–13. doi: 10.1007/s10388-026-01222-2 (PMC13723658; doi:10.1007/s10388-026-01222-2)
Supplement: Supplementary file 1 [file 10388_2026_1222_MOESM1_ESM.docx]

**Title:** Potassium-competitive acid blockers and proton-pump inhibitors for the healing and maintenance of erosive esophagitis: a comprehensive and updated systematic review and network meta-analysis

**Authors:** Daniel Martin Simadibrata^1^, Thai Hau Koo^2^, Raúl Alberto Jiménez-Castillo^2^, Ronnie Fass^2^

**Supplementary Files**

**Supplementary File 1. PRISMA NMA Checklist of Items for Systematic Reviews Involving Network Meta-Analysis**

**Supplementary File 2. Search Strategy Details to February 2, 2025 (Updated to June 25, 2025)**

**Supplementary File 3. Baseline Characteristics of Participants Included in Randomized Controlled Trials Evaluating Potassium-Competitive Acid Blocker and Proton Pump Inhibitor for Healing of Erosive Esophagitis**

**Supplementary File 4. Eligibility Criteria of Included Randomized Controlled Trials Evaluating Potassium-Competitive Acid Blocker and Proton Pump Inhibitor for Healing of Erosive Esophagitis**

**Supplementary File 5. Baseline Characteristics of Participants Included in Randomized Controlled Trials That Evaluating Potassium-Competitive Acid Blocker and Proton Pump Inhibitor for Maintenance of Healing of Erosive Esophagitis**

**Supplementary File 6. Eligibility Criteria of Included Randomized Controlled Trials Evaluating Potassium-Competitive Acid Blocker and Proton Pump Inhibitor for Maintenance of Healing of Erosive Esophagitis**

**Supplementary File 7. Risk of Bias Assessment According to the Cochrane ROB2 Tool for Randomized Controlled Trials Evaluating the Healing of Erosive Esophagitis**

**Supplementary File 8. Risk of Bias Assessment According to the Cochrane ROB2 Tool for Randomized Controlled Trials Evaluating the Healing of Erosive Esophagitis**

**Supplementary File 9. Comparison-adjusted Funnel Plot for Healing of Erosive Esophagitis at Week 4. Egger’s test p-value = 0.672.**

**Supplementary File 10. League Table for Healing of EE at Week 4.**

**Supplementary Table 11. Comparison-adjusted Funnel Plot for Healing of Erosive Esophagitis at Week 8. Egger’s test p-value = 0.735.**

**Supplementary File 12. League Table for Healing of EE at Week 8.**

**Supplementary File 13. Network Meta-Analysis illustrating the healing rates of potassium-competitive acid blockers (PCAB) and proton pump inhibitors (PPI) at 2 weeks**. All treatments are compared against vonoprazan 20 mg once daily (QD) as the reference standard and ranked according to their P-score. Higher P-scores indicate superior healing efficacy.

**Supplementary File 14. Comparison-adjusted Funnel Plot for Healing of Erosive Esophagitis at Week 2. Egger’s test p-value = 0.957.**

**Supplementary File 15. League Table for Healing of EE at Week 2.**

**Supplementary File 16. Network Meta-Analysis illustrating the healing rates of potassium-competitive acid blockers (PCAB) and proton pump inhibitors (PPI) at (A) 2 weeks, (B) 4 weeks, and (C) 8 weeks for erosive esophagitis patients with Los Angeles Grade A/B equivalent (Mild/Moderate).** All treatments are compared against vonoprazan 20 mg once daily (QD) as the reference standard and ranked according to their P-score. Higher P-scores indicate superior healing efficacy.

**Supplementary File 17. Network Meta-Analysis illustrating the healing rates of potassium-competitive acid blockers (PCAB) and proton pump inhibitors (PPI) at (A) 2 weeks, (B) 4 weeks, and (C) 8 weeks for erosive esophagitis patients with Los Angeles Grade C/D equivalent (Severe).** All treatments are compared against vonoprazan 20 mg once daily (QD) as the reference standard and ranked according to their P-score. Higher P-scores indicate superior healing efficacy.

**Supplementary File 18. Network Meta-Analysis illustrating the healing rates of potassium-competitive acid blockers (PCAB) and proton pump inhibitors (PPI) at (A) 2 weeks, (B) 4 weeks, and (C) 8 weeks for erosive esophagitis patients with Los Angeles Grade B-D equivalent.** All treatments are compared against vonoprazan 20 mg once daily (QD) as the reference standard and ranked according to their P-score. Higher P-scores indicate superior healing efficacy**.**

**Supplementary File 19. Comparison-adjusted Funnel Plot for Any Adverse Events in Randomized Controlled Trials Evaluating Healing of Erosive Esophagitis. Egger’s test p-value = 0.939**

**Supplementary File 20. Comparison-adjusted Funnel Plot for Serious Adverse Events in Randomized Controlled Trials Evaluating Healing of Erosive Esophagitis. Egger’s test p-value = 0.301**

**Supplementary File 21. League Table for Maintenance of Healed EE at 6 Months.**

**Supplementary File 22. Comparison-adjusted Funnel Plot for Maintenance of Healing of Erosive Esophagitis at 6 months. Egger’s test p-value = 0.439.**

**Supplementary File 23. Network Meta-Analysis of maintenance of healing rates of potassium-competitive acid blockers (PCAB) and proton pump inhibitors (PPI) at 6 months based on Los Angeles (A) Grade A/B equivalent, (B) Grade C/D Equivalent, and (C) Grade B-D equivalent**. Vonoprazan 10 mg once daily (QD) is the reference treatment. Treatments are ranked based on their P-scores, where higher scores denote improved rates of non-recurrence.

**Supplementary File 24. League Table for Maintenance of Healed EE at 3 Months.**

**Supplementary File 25. Comparison-adjusted Funnel Plot for Maintenance of Healing of Erosive Esophagitis at 3 months. Egger’s test p-value = 0.609.**

**Supplementary File 26. League Table for Maintenance of Healed EE at 12 Months.**

**Supplementary File 27. Comparison-adjusted Funnel Plot for Maintenance of Healing of Erosive Esophagitis at 12 months. Egger’s test p-value = 0.619.**

**Supplementary File 28. Comparison-adjusted Funnel Plot for Any Adverse Events in Randomized Controlled Trials Evaluating Maintenance of Healing of Erosive Esophagitis. Egger’s test p-value = 0.335.**

**Supplementary File 29. Comparison-adjusted Funnel Plot for Any Adverse Events in Randomized Controlled Trials Evaluating Maintenance of Healing of Erosive Esophagitis. Egger’s test p-value = 0.448.**

**Supplementary File 1. PRISMA NMA Checklist of Items for Systematic Reviews Involving Network Meta-Analysis**

| **Section/Topic** | **Item #** | **Checklist Item** | **Reported on Page #** |
| --- | --- | --- | --- |
| **TITLE** |  |  |  |
| Title | 1 | Identify the report as a systematic review *incorporating a network meta-analysis (or related form of meta-analysis).* | ***1*** |
| **ABSTRACT** |  |  |  |
| Structured summary | 2 | Provide a structured summary including, as applicable:  **Background:** main objectives  **Methods:** data sources; study eligibility criteria, participants, and interventions; study appraisal; and *synthesis methods, such as network meta-analysis.*  **Results:** number of studies and participants identified; summary estimates with corresponding confidence/credible intervals; *treatment rankings may also be discussed. Authors may choose to summarize pairwise comparisons against a chosen treatment included in their analyses for brevity.*  **Discussion/Conclusions:** limitations; conclusions and implications of findings.  **Other:** primary source of funding; systematic review registration number with registry name. | 2 |
| **INTRODUCTION** |  |  |  |
| Rationale | 3 | Describe the rationale for the review in the context of what is already known*, including mention of why a network meta-analysis has been conducted.* | ***3-4*** |
| Objectives | 4 | Provide an explicit statement of questions being addressed, with reference to participants, interventions, comparisons, outcomes, and study design (PICOS). | 4 |
| **METHODS** |  |  |  |
| Protocol and registration | 5 | Indicate whether a review protocol exists and if and where it can be accessed (e.g., Web address); and, if available, provide registration information, including registration number. | 5 |
| Eligibility criteria | 6 | Specify study characteristics (e.g., PICOS, length of follow-up) and report characteristics (e.g., years considered, language, publication status) used as criteria for eligibility, giving rationale. *Clearly describe eligible treatments included in the treatment network, and note whether any have been clustered or merged into the same node (with justification).* | ***5*** |
| Information sources | 7 | Describe all information sources (e.g., databases with dates of coverage, contact with study authors to identify additional studies) in the search and date last searched. | 5 |
| Search | 8 | Present full electronic search strategy for at least one database, including any limits used, such that it could be repeated. | 5, suppl |
| Study selection | 9 | State the process for selecting studies (i.e., screening, eligibility, included in systematic review, and, if applicable, included in the meta-analysis). | 5-6 |
| Data collection process | 10 | Describe method of data extraction from reports (e.g., piloted forms, independently, in duplicate) and any processes for obtaining and confirming data from investigators. | 6 |
| Data items | 11 | List and define all variables for which data were sought (e.g., PICOS, funding sources) and any assumptions and simplifications made. | 6-7 |
| **Geometry of the network** | **S1** | Describe methods used to explore the geometry of the treatment network under study and potential biases related to it. This should include how the evidence base has been graphically summarized for presentation, and what characteristics were compiled and used to describe the evidence base to readers. | ***NA*** |
| Risk of bias within individual studies | 12 | Describe methods used for assessing risk of bias of individual studies (including specification of whether this was done at the study or outcome level), and how this information is to be used in any data synthesis. | 6-7 |
| Summary measures | 13 | State the principal summary measures (e.g., risk ratio, difference in means). *Also describe the use of additional summary measures assessed, such as treatment rankings and surface under the cumulative ranking curve (SUCRA) values, as well as modified approaches used to present summary findings from meta-analyses.* | 7 |
| Planned methods of analysis | 14 | Describe the methods of handling data and combining results of studies for each network meta-analysis. This should include, but not be limited to:   - *Handling of multi-arm trials;* - *Selection of variance structure;* - *Selection of prior distributions in Bayesian analyses; and* - *Assessment of model fit.* | 7 |
| **Assessment of Inconsistency** | **S2** | Describe the statistical methods used to evaluate the agreement of direct and indirect evidence in the treatment network(s) studied. Describe efforts taken to address its presence when found. | NA |
| Risk of bias across studies | 15 | Specify any assessment of risk of bias that may affect the cumulative evidence (e.g., publication bias, selective reporting within studies). | **7** |
| Additional analyses | 16 | Describe methods of additional analyses if done, indicating which were pre-specified. This may include, but not be limited to, the following:   - Sensitivity or subgroup analyses; - Meta-regression analyses; - *Alternative formulations of the treatment network; and* - *Use of alternative prior distributions for Bayesian analyses (if applicable).* | ***7*** |
| **RESULTS†** |  |  |  |
| Study selection | 17 | Give numbers of studies screened, assessed for eligibility, and included in the review, with reasons for exclusions at each stage, ideally with a flow diagram. | 8 |
| **Presentation of network structure** | **S3** | Provide a network graph of the included studies to enable visualization of the geometry of the treatment network. | ***NA*** |
| **Summary of network geometry** | **S4** | Provide a brief overview of characteristics of the treatment network. This may include commentary on the abundance of trials and randomized patients for the different interventions and pairwise comparisons in the network, gaps of evidence in the treatment network, and potential biases reflected by the network structure. | ***NA*** |
| Study characteristics | 18 | For each study, present characteristics for which data were extracted (e.g., study size, PICOS, follow-up period) and provide the citations. | 8, suppl |
| Risk of bias within studies | 19 | Present data on risk of bias of each study and, if available, any outcome level assessment. | 8, suppl |
| Results of individual studies | 20 | For all outcomes considered (benefits or harms), present, for each study: 1) simple summary data for each intervention group, and 2) effect estimates and confidence intervals. *Modified approaches may be needed to deal with information from larger networks.* | 8-12, suppl |
| Synthesis of results | 21 | Present results of each meta-analysis done, including confidence/credible intervals. *In larger networks, authors may focus on comparisons versus a particular comparator (e.g. placebo or standard care), with full findings presented in an appendix. League tables and forest plots may be considered to summarize pairwise comparisons.* If additional summary measures were explored (such as treatment rankings), these should also be presented. | 8, suppl |
| **Exploration for inconsistency** | **S5** | Describe results from investigations of inconsistency. This may include such information as measures of model fit to compare consistency and inconsistency models, *P* values from statistical tests, or summary of inconsistency estimates from different parts of the treatment network. | ***NA*** |
| Risk of bias across studies | 22 | Present results of any assessment of risk of bias across studies for the evidence base being studied. | 8-12 |
| Results of additional analyses | 23 | Give results of additional analyses, if done (e.g., sensitivity or subgroup analyses, meta-regression analyses*, alternative network geometries studied, alternative choice of prior distributions for Bayesian analyses,* and so forth). | ***8-12*** |
| **DISCUSSION** |  |  |  |
| Summary of evidence | 24 | Summarize the main findings, including the strength of evidence for each main outcome; consider their relevance to key groups (e.g., healthcare providers, users, and policy-makers). | 13-15 |
| Limitations | 25 | Discuss limitations at study and outcome level (e.g., risk of bias), and at review level (e.g., incomplete retrieval of identified research, reporting bias). *Comment on the validity of the assumptions, such as transitivity and consistency. Comment on any concerns regarding network geometry (e.g., avoidance of certain comparisons).* | 16 |
| Conclusions | 26 | Provide a general interpretation of the results in the context of other evidence, and implications for future research. | 16 |
| **FUNDING** |  |  |  |
| Funding | 27 | Describe sources of funding for the systematic review and other support (e.g., supply of data); role of funders for the systematic review. This should also include information regarding whether funding has been received from manufacturers of treatments in the network and/or whether some of the authors are content experts with professional conflicts of interest that could affect use of treatments in the network. | ***1*** |

**Supplementary File 2. Search Strategy Details to February 2, 2025 (Updated to December 15, 2025)**

**Database: Embase <1974 to December 15, 2025>**
**Search Strategy:**
**1**  ("erosive esophagitis" or "erosive oesophagitis" or "reflux esophagitis" or "reflux oesophagitis").mp. (16391)
**2**  reflux esophagitis/ (19685)
**3**  1 or 2 (17392)
**4**  ("Proton pump inhibitor*" or PPI* or omeprazole or lansoprazole or esomeprazole or pantoprazole or dexlansoprazole or rabeprazole or tenatoprazole or ilaprazole).mp. (173214)
**5**  proton pump inhibitor/ or omeprazole/ or lansoprazole/ or esomeprazole/ or pantoprazole/ or dexlansoprazole/ or rabeprazole/ or ilaprazole/ (115170)
**6**  4 or 5 (173214)
**7**  ("Potassium competitive acid blocker*" or PCAB* or vonoprazan or tak-438 or takecab or soraprazan or revaprazan or YH1885 or linaprazan or AZD0865 or tegoprazan or keverprazan or fexuprazan or zastaprazan).mp. (3116)
**8**  potassium competitive acid blocker/ or vonoprazan/ or fexuprazan/ or soraprazan/ or tegoprazan/ or linaprazan/ or revaprazan/ (2702)
**9**  7 or 8 (3116)
**10**  6 or 9 (174292)
**11**  3 and 10 (6431)
**12**  exp randomized controlled trial/ (1129365)
**13**  controlled clinical trial/ (460992)
**14**  random$.ti,ab. (2545540)
**15**  randomization/ (101717)
**16**  intermethod comparison/ (318072)
**17**  placebo.ti,ab. (470719)
**18**  (compare or compared or comparison).ti,ab. (8953219)
**19**  ((evaluated or evaluate or evaluating or assessed or assess) and (compare or compared or comparing or comparison)).ab. (3417704)
**20**  (open adj label).ti,ab. (192425)
**21**  ((double or single or doubly or singly) adj (blind or blinded or blindly)).ti,ab. (373058)
**22**  double blind procedure/ (314276)
**23**  parallel group$1.ti,ab. (53923)
**24**  (crossover or cross over).ti,ab. (158551)
**25**  ((assign$ or match or matched or allocation) adj5 (alternate or group$1 or intervention$1 or patient$1 or subject$1 or participant$1)).ti,ab. (522448)
**26**  (assigned or allocated).ti,ab. (620956)
**27**  (controlled adj7 (study or design or trial)).ti,ab. (649198)
**28**  (volunteer or volunteers).ti,ab. (325717)
**29**  human experiment/ (737458)
**30**  trial.ti. (575149)
**31**  or/12-30 (11864228)
**32**  (random$ adj sampl$ adj7 ("cross section$" or questionnaire$1 or survey$ or database$1)).ti,ab. not (comparative study/ or controlled study/ or randomi?ed controlled.ti,ab. or randomly assigned.ti,ab.) (10842)
**33**  cross-sectional study/ not (exp randomized controlled trial/ or controlled clinical trial/ or controlled study/ or randomi?ed controlled.ti,ab. or control group$1.ti,ab.) (482154)
**34**  ((case adj control$).mp. and random$.ti,ab.) not randomi?ed controlled.ti,ab. [mp=title, abstract, heading word, drug trade name, original title, device manufacturer, drug manufacturer, device trade name, keyword heading word, floating subheading word, candidate term word] (30473)
**35**  systematic review.ti,ab. not (trial or study).ti. (437733)
**36**  (non random$ not random$).ti,ab. (0)
**37**  "random field$".ti,ab. (3272)
**38**  (random cluster adj3 sample$).ti,ab. (238)
**39**  (review.ab. and review.pt.) not trial.ti. (1355576)
**40**  "we searched".ab. and (review.ti. or review.pt.) (61253)
**41**  "update review".ab. (156)
**42**  (databases adj4 searched).ab. (81073)
**43**  (rat or rats or mouse or mice or swine or porcine or murine or sheep or lambs or pigs or piglets or rabbit or rabbits or cat or cats or dog or dogs or cattle or bovine or monkey or monkeys or trout or marmoset$1).ti. and animal experiment/ (1329260)
**44**  animal experiment/ not (human experiment/ or human/) (2812362)
**45**  or/32-44 (5058731)
**46**  31 not 45 (10373194)
**47**  11 and 46 (**2260**)

**Database: Ovid MEDLINE(R) and Epub Ahead of Print, In-Process & Other Non-Indexed Citations and Ovid MEDLINE(R) Daily 1946 to December 15, 2025**
**Search Strategy:**
**1**  ("erosive esophagitis" or "erosive oesophagitis" or "reflux esophagitis" or "reflux oesophagitis").mp. (6468)
**2**  Esophagitis/ (14292)
**3**  1 or 2 (17301)
**4**  ("Proton pump inhibitor*" or PPI* or omeprazole or lansoprazole or esomeprazole or pantoprazole or dexlansoprazole or rabeprazole or tenatoprazole or ilaprazole).mp. (74982)
**5**  Proton Pump Inhibitors/ or Omeprazole/ or Lansoprazole/ or Esomeprazole/ or Pantoprazole/ or Dexlansoprazole/ or Rabeprazole/ (22984)
**6**  4 or 5 (74982)
**7**  ("Potassium competitive acid blocker*" or PCAB* or vonoprazan or tak-438 or takecab or soraprazan or revaprazan or YH1885 or linaprazan or AZD0865 or tegoprazan or keverprazan or fexuprazan or zastaprazan).mp. (1446)
**8**  6 or 7 (74982)
**9**  3 and 8 (3134)
**10**  exp randomized controlled trial/ (655260)
**11**  controlled clinical trial.pt. (95777)
**12**  randomized.ab. (724439)
**13**  placebo.ab. (265175)
**14**  drug therapy.fs. (2883853)
**15**  randomly.ab. (476215)
**16**  trial.ab. (792170)
**17**  groups.ab. (2961436)
**18**  10 or 11 or 12 or 13 or 14 or 15 or 16 or 17 (6498607)
**19**  exp animals/ not humans.sh. (5406059)
**20**  18 not 19 (5703909)
**21**  9 and 20 (**2187**)

**Cochrane Library to December 15, 2025**
ID	Search	Hits
#1	("erosive esophagitis" or "erosive oesophagitis" or "reflux esophagitis" or "reflux oesophagitis"):ti,ab,kw (Word variations have been searched)	1707
#2	MeSH descriptor: [Esophagitis] explode all trees (Word variations have been searched)	1112
#3	#1 or #2	2257
#4	(omeprazole or lansoprazole or esomeprazole or pantoprazole or dexlansoprazole or rabeprazole or tenatoprazole or ilaprazole):ti,ab,kw (Word variations have been searched)	9615
#5	("Proton pump inhibitor" or PPI*):ti,ab,kw (Word variations have been searched)	7339
#6	MeSH descriptor: [Proton Pump Inhibitors] explode all trees	2043
#7	MeSH descriptor: [Omeprazole] explode all trees	3538
#8	MeSH descriptor: [Lansoprazole] explode all trees	925
#9	MeSH descriptor: [Esomeprazole] explode all trees	964
#10	MeSH descriptor: [Pantoprazole] explode all trees	628
#11	MeSH descriptor: [Dexlansoprazole] explode all trees	223
#12	MeSH descriptor: [Rabeprazole] explode all trees	581
#13	#4 or #5 or #6 or #7 or #8 or #9 or #10 or #11 or #12	13471
#14	("Potassium competitive acid blocker" or PCAB*):ti,ab,kw (Word variations have been searched)	368
#15	(vonoprazan or tak-438 or takecab or soraprazan or revaprazan or YH1885 or linaprazan or AZD0865 or tegoprazan or keverprazan or fexuprazan or zastaprazan):ti,ab,kw (Word variations have been searched)	897
#16	#14 or #15	970
#17	#13 or #16	13786
#18	#3 and #17	**1137**

**Supplementary File 3. Baseline Characteristics of Participants Included in Randomized Controlled Trials Evaluating Potassium-Competitive Acid Blocker and Proton Pump Inhibitor for Healing of Erosive Esophagitis**

**Supplementary File 4. Eligibility Criteria of Included Randomized Controlled Trials Evaluating Potassium-Competitive Acid Blocker and Proton Pump Inhibitor for Healing of Erosive Esophagitis**

| **No** | **Author (Year)** | **Trial Registration** | **Country** | **Center** | **Inclusion Criteria** | **Exclusion Criteria** |
| --- | --- | --- | --- | --- | --- | --- |
| **1** | Adachi 2003 | NR | Japan | Multicenter | Patients with endoscopically proven reflux esophagitis. None of the patients had undergone gastric or antireflux surgery, or H pylori eradication therapy. | Patients with active peptic ulcer, upper GI cancers, malignant diseases of other organs, severe cardiac, hepatic, or renal diseases, anemia (Hb <10 g/dL), or who were pregnant and/or lactating. |
| **2** | Ashida 2015 | JapicCTI-090928 | Japan | Multicenter | Patients with endoscopically confirmed EE (LA grades A-D) and were aged 20 years or older. | Patients with enrolment in a previous clinical trial using VPZ; presence of coexisting diseases of the esophagus (e.g. EoE, esophageal varices, scleroderma, viral or fungal infection and esophageal stenosis); a history of radiation therapy or cryotherapy to the esophagus; presence of caustic or physiochemical esophageal trauma; a history of surgery or any treatment that could induce gastroesophageal reflux or affect the stomach or duodenum (excluding endoscopic removal of benign polyps); presence of acute upper GI hemorrhage or a gastric/duodenal ulcer (active stage A1/A2 or healing stage H2) within 30 days prior to visit 1 (with the exception of gastric or duodenal erosions); a history of ZES or any of its complications or any other gastric acid hypersecretory disorder; a history of hypersensitivity or allergy to VPZ (or any of its pharmaceutical excipients) or PPIs; presence of complications of any significant neurological, cardiovascular, pulmonary, hepatic, renal, metabolic, GI, urological, endocrinological or hematological disorder; a history of malignancy (except for basal cell carcinoma of the skin) within 3 years prior; treatment with any PPI within 14 days or any H2RA within 7 days; or abnormal laboratory test results, i.e. serum Cr >2 mg/dL, ALT or AST >2x ULN, ALT or AST level >1.5x ULN and total bilirubin level >2 mg/dL. |
| **3** | Ashida 2016 | NCT01452698 | Japan | Multicenter | Male or female outpatients aged ≥20 years with endoscopically confirmed EE LA Classification Grades A-D. | Patients with complications associated with esophagitis, acute upper GI bleeding; history of hypersecretion disorders; surgery or treatment affecting GERD; serious organ disorders; requiring surgery; history of drug abuse; AIDS or hepatitis; history of malignancy; abnormal laboratory test values; pregnant female subjects; concomitant use of medicines affecting the study. |
| **4** | Bardhan 1995 | NR | UK | Multicenter | Patients >18 years, with a history of typical reflux symptoms, who had endoscopically proven esophagitis. | Patients with esophageal stricture or other significant GI disease, those with previous esophageal or gastroduodenal surgery, patients with major coexistent systemic disease, pregnant women, and those who had taken specific ulcer healing therapy for 6 days prior to entry. |
| **5** | Bardhan 2001 | NR | UK | Multicenter | Patients aged >18 years old, SM grade I, symptoms acid eructation and/or heartburn and/or pain on swallowing within 3 days of entry. | Patients with grade II, III or IV reflux esophagitis, pregnant and nursing women or women of child bearing age who had not used effective methods of contraception for at least 3 months prior to the start of the study, patients with malignant or any other severe diseases, patients with clinically relevant deviations from the normal range in laboratory values as assessed by the investigator and patients with a history of ZES, pyloric stenosis, peptic ulcer or ulcer complications and previous surgery of the esophagus and/or GI tract (except for appendectomy, cholecystectomy and polypectomy). Patients were excluded if they had received antacids, or drugs which might potentially interact with either of the study drugs, had taken substituted benzimidazoles or H2RA for more than 3 days in the 20 days prior to the start of the study. |
| **6** | Castell 1996 | NR | NR | Multicenter | Adult patients with erosive reflux esophagitis ≥.grade 2. | Patients with duodenal ulcer and/or gastric ulcer ≥3 mm in diameter; Barrett’s esophagus displaying dysplastic changes, esophagitis due to a coexisting systemic disease, ZES, esophageal varices, or malignancy necessitating active treatment (except basal cell carcinoma), a history of gastric, duodenal, or esophageal surgery (except simple ulcer oversew), esophageal stricture necessitating dilation, or a history of stricture dilation within the previous 12 weeks; current alcohol abuse, illegal drug use, or drug abuse within the past 12 months, chronic use of any ulcerogenic drug within 30 days before the screening endoscopy, chronic anticoagulant therapy, consumption of omeprazole or participation in any other experimental drug study within the previous 12 week, or participation in any other lansoprazole study. |
| **7** | Castell 2002 | NR | US | Multicenter | Patients aged 18-75 years, with endoscopically confirmed EE (LA classification grades A–D) within 1 week of randomization and heartburn (≥2 days during the 7 days before baseline). Women had to be nonpregnant, nonlactating, or using approved contraception. | Patients with any bleeding disorder or signs of GI bleeding at the time of baseline EGD or within 3 days before randomization or had a history of gastric or esophageal surgery, ZES, primary motility disorders, esophageal stricture, or Barrett’s esophagus ≥3 cm, evidence of upper GI malignancy, or severe comorbid disease, received PPI within 28 days or used H2RA within 2 weeks before baseline visit, or received other concomitant medication that might affect the interpretation of the treatment outcome (quinidine, diazepam, diphenylhydantoins, mephenytoin, warfarin, anticholinergics, prostaglandin analogs, antineoplastic agents, salicylates) and those with known hypersensitivity to any component of esomeprazole, lansoprazole, or Gelusil. |
| **8** | Chen 2005 | NR | Taiwan | Single center | Outpatients who sought medical care because of typical GERD symptoms for at least 1 month who received an endoscopy to confirm the EE diagnosis according to LA grading system. | Patients with coexistence of healed or active peptic ulcer, GI malignancy, esophagogastric surgical history, esophagitis obviously resulted from systemic diseases, infections, drugs, burn, radiotherapy or physical deformity, severe esophageal stricture requiring dilatation at first endoscopy or expectation of requiring dilatation during the study, recent PPI treatment within 8 days prior to endoscopy, or using PPIs for more than 5 days in the last 28 days prior to endoscopy, with H pylori eradication therapy within the last 28 days prior to randomization or at any time during the study, using other antisecretory or prokinetic agents between endoscopy and randomization or at any time during the study, or with any investigational (non-approved) drug during the last 30 days prior to randomization. |
| **9** | Chen 2022 | ChiCTR2100043456 | China | Multicenter | Patients with endoscopically confirmed EE (LA grades A-D) within 7 days of randomisation and aged 18-75 years old. | Patients with prior usage of keverprazan, unable to receive upper GI endoscopy, allergic to keverprazan or lansoprazole, endoscopically confirmed organic esophageal diseases except esophagitis, hiatus hernia, or Barrett’s esophagus <3 cm, with rheumatic immune diseases that might affect esophageal motility, who received surgery or therapy which might affect gastric acid secretion or influence structure or function of the upper digestive tract, history of malignant tumor within 5 years prior to screening, ALT or AST >1.5× ULN or serum Cr >ULN, use of NSAIDs, systemic glucocorticoids, antiplatelet drugs (such as aspirin and clopidogrel), anticoagulants, atazanavir sulfate or rilpivirine and could not discontinue the drugs during the study, received PPI, PCAB, H2RA for GERD treatment within 7 days prior to randomisation, had a history of drug or alcohol abuse, female subjects who were in or preparing for pregnancy or lactation. |
| **10** | Cho 2012 | NR | Korea | Multicenter | Patients with symptomatic GERD aged 18–75 years who had endoscopically proven reflux esophagitis. | Patients who had any planned surgery during the course of the trial; evidence of uncontrolled systemic disease; need for continuous anticoagulant, H2RA, sucralfate, misoprostol, prokinetics, or non-approved antacids at any time during the trial; use of drugs with significant anticholinergic effects; or chronic use ([12 doses per NSAIDs, including selective cyclooxygenase 2 inhibitors. |
| **11** | Cloud 1998 | NR | US | Multicenter | Men and women over the age of 18 years who met modified HD grade 2-4 esophagitis on endoscopic examination. | Women of childbearing potential who were not using an approved method of contraception, as were pregnant or lactating women; with a history of esophageal, gastric, or definitive acid-lowering surgery (except single closure of perforation); pyloric stenosis; concurrent serious systemic disorders (renal or hepatic insufficiency or cancer); current treatment with ulcerogenic drug regimens; treatment with therapeutic doses of an antireflux agent (eg, H2RA, PPI, prostaglandin, or sucralfate) for more than 5 days within 2 weeks prior to enrollment; treatment with an investigational agent within the last 30 days; any condition associated with poor patient compliance (eg, drug or alcohol abuse); or abnormal laboratory values on admission considered clinically significant by the investigator; undergoing treatment with antibiotics or bismuth; active ulceration other than the primary study condition. |
| **12** | Cordova-Villalobos 1996 | NR | Mexico | Single Center | Patients between 18 and 65 years of age with reflux esophagitis grades II to IV according to the LA classification and with a least one of the following three symptoms: heartburn, acid regurgitation, pain on swallowing. | Patients with severe disease of any organ, cancer, ZES, undergone esophageal or gastric surgery, pyloric stenosis, suspected allergy to PPI, drug ingestion that could interact with PPI, habitual alcoholics, pregnant women or women who did not use effective contraceptive methods for at least three months prior to the start of treatment. |
| **13** | Corinaldesi 1995 | NR | Belgium, France, Italy, and Netherlands | Multicenter | Patients of either sex, aged 18 years or older, with endoscopically diagnosed reflux esophagitis grade II or III according to SM criteria and at least one of the symptoms: acid regurgitation, heartburn or pain on swallowing. | Patients with peptic ulcer, reflux esophagitis grades I or IV, any severe concomitant disease, consumption of glucocorticoids, NSAIDs, pH-dependent absorption, drugs which may interact with substituted benzimidazoles, and concomitant supportive medication e.g. antacids. |
| **14** | Dekkers 1999 | NR | Europe | Multicenter | Men and women of 18 years of age or older with a history of GERD of at least 3 months prior to enrolling in the study and, at admission, endoscopic evidence of erosive or ulcerative esophagitis (Grade ≥2, when measure by modified HD grading scale); women if they were not of childbearing potential or were using an approved method of contraception. | Patients with primary esophageal stricture or esophagitis secondary to systemic events such as scleroderma or irritant ingestion (however, patients with Barrett’s esophagus could be included, provided that esophagitis grading criteria were met), historical evidence of esophageal motility disorders, a history of definitive acid-lowering surgery or previous esophageal or gastric surgery (except for simple closure of perforations), esophageal and/or gastric varices, pyloric stenosis, treatment with full therapeutic doses of an H2RA, prostaglandin or sucralfate for more than 5 consecutive days within 2 weeks prior to enrolling in the study, treatment with therapeutic doses of a PPI for 3 consecutive days within 2 weeks prior to enrolment and any treatment with therapeutic PPI within 5 days to enrolment, and concurrent treatment with high doses of corticosteroids, NSAIDs, anticoagulants, anticholinergics, antidepressants, motility agents, antineoplastics or antiepileptics; presented with active peptic ulceration, endoscopic evidence of active GI bleeding or had known ZES. |
| **15** | Delchier 2000 | NR | Europe | Multicenter | Male and female outpatients over the age of 18 with a history of symptomatic GERD over a 3-month period prior to enrolling in the study and endoscopically confirmed ulcerative or erosive GERD (grade 2 or above according to the modified HD grading scale). | Patients with a normal esophagus or grade 1 esophagitis as determined by endoscopy, an esophageal stricture that restricted the passage of the endoscope, esophagitis secondary to systemic events, or a primary esophageal motility disorder; prior esophageal or gastric surgery, esophageal or gastric varices, significant pyloric stenosis or recent treatment with therapeutic doses of a PPI. |
| **16** | Dupas 2001 | NR | France | Multicenter | Patients aged 18 year or above, of both sexes, with symptoms: heartburn, acid regurgitation, pain on swallowing and proven grade II and III reflux esophagitis (according to SM classification). | Patients with duodenal or gastric ulcer, ZES, grades <II or >III esophagitis, infectious, caustic or post-radiotherapy esophagitis, surgical history of the esophagus, stomach or duodenum except for basal peptic ulcer suture, any serious, severe or malignant disease susceptible to interfere with the study outcome, intake of substituted benzimidazoles for more than 3 days within the 20 days prior to the start of the study, regular intake of oral anticoagulants, phenytoin, theophylline, glucocorticosteroids, NSAIDs or aspirin, simultaneous intake of drugs whose absorption is pH-dependent (e.g., ketoconazole), women of childbearing age without reliable contraception for at least 3 months, pregnant or nursing women and women under hormonal contraception, any evidence of alcohol or drug abuse and clinically relevant deviations from the normal range in the following laboratory parameters: Cr >300 umol/L, AST and/or ALT >2x the normal range. |
| **17** | Earnest 1998 | NR | US | Multicenter | Patients with endoscopically confirmed reflux esophagitis of at least grade 2 severity within 7 days of entering the treatment phase of the study. | Patients with any other serious concomitant medical illness, if they used NSAIDs or aspirin, or if they had taken any form of ulcer medication excluding antacids in the preceding 8 weeks, a current history of alcohol or drug abuse, women who were lactating, pregnant, or at risk of becoming pregnant. |
| **18** | Fennerty 2005 | D9612L00046/Study 322 | US | Multicenter | Men and non-pregnant, non-lactating women between the ages of 18 and 75 years with LA grades C or D EE confirmed by an EGD within 1 week before randomization and heartburn (described as a burning feeling, rising from the stomach or lower part of the chest up towards the neck) for ≥2 of the 7 days before randomization. | Patients with any bleeding disorder or signs of GI bleeding at the time of baseline endoscopy or within 3 days before randomization; had a history of gastric or esophageal surgery, except for simple closure of perforated ulcer; or had current or historical evidence (within 3 months) of ZES, primary esophageal motility disorders (achalasia, scleroderma and/or primary esophageal spasm), IBD, pancreatitis, malabsorption, generalized bleeding disorders resulting from hemorrhagic diathesis, esophageal stricture, duodenal ulcer, gastric ulcer, evidence of upper GI malignancy, Barrett’s esophagus (>3 cm), significant dysplastic changes in the esophagus, or any other severe concomitant disease. Patients who used a PPI within 28 days before the baseline visit or daily H2RA in doses exceeding standard approved prescription-strength doses within the last 3 months were excluded, as were patients who had need for continuous concurrent therapy with warfarin or other anticoagulants, prostaglandin analogues, antineoplastic agents, salicylates (unless ≤165 mg daily for cardiovascular prophylaxis), steroids (oral or intravenous), pro-motility drugs, sucralfate, NSAID, phenytoin, or tegaserod. Use of H pylori eradication therapy or a concomitant medication that depends on the presence of gastric acid for optimal absorption was not permitted. |
| **19** | Gillessen 2004 | NR | Germany | Multicenter | Patients who are endoscopically proven GERD (LA grade B and C) and typical symptoms of GERD (heartburn, acid regurgitation, and dysphagia). | Patients with endoscopically proven LA Grade A or D GERD, peptic ulcer complications (Forrest Ia, Ib, IIa, and IIb), florid peptic ulcer disease, a medical history of ZES, pyloric stenosis, prior esophageal and/or GI surgery (with the exception of appendectomy, cholecystectomy, or polypectomy), known allergies, especially to any of the 2 study drugs and their components, rare genetic diseases (such as fructose intolerance, glucose–galactose malabsorption, sac- charose–isomaltose deficiency), severe concomitant diseases, malignant diseases within the past 5 years, moderate to severe malfunctions of liver and kidney disease, clinically relevant deviations from normal laboratory parameters or a history of alcohol or drug abuse, are pregnant or considered using ineffective contraception, used systemic glucocorticoids or NSAIDs on more than 3 successive days within the last 28 days prior to the initiation of the study, used PPIs within the last 14 days, H2RA, or prokinetics during the last 10 days before start of the study, intake of sucralfate and antacids within the last 3 days before study start, or intake of ketoconazole during the course of study. |
| **20** | Hatlebakk 1993 | NR | Norway and Sweden | Multicenter | Patients presenting with grade 1 and 2 esophagitis. | Patients presenting with grade 1 and 2 esophagitis were included in separate strata, whereas patients with grade 3 lesions or Barrett’s esophagus (defined as metaplasia extending more than 3 cm proximal from the cardia) were excluded from the study. Patients with active peptic ulcer disease, inflammatory bowel disease, malignant disease in any organ or previous surgery of the upper GI tract or active biliary or pancreatic disease were excluded from entering the study. |
| **21** | Hetzel 1988 | NR | Australia | Multicenter | Outpatients with erosive or ulcerative esophagitis of grade 2 or greater. | Patients aged <18 years or >80 years, previous esophagogastric surgery (other than failed antireflux surgery); esophagitis associated with systemic disease (e.g., scleroderma); esophagitis due to viral or fungal infection or caustic, radiation, or other physicochemical trauma; concurrent gastric ulcer, duodenal ulcer, pyloric stenosis, or malignancy; ongoing GI hemorrhage; other serious illness (cardiac, renal, or hepatic disease); other investigational drug use within 4 week of the initial endoscopy or use of any other antisecretory or prokinetic medication after the initial endoscopy; or alcoholism, drug abuse, or other disorders considered to prevent compliance with treatment, pregnant or lactating women. |
| **22** | Howden 2002 | NR | US | Multicenter | Patients who were at least 18 years of age with erosive esophagitis of at least grade 2 confirmed by endoscopy performed within 7 days of study. | Patients with active duodenal or gastric ulcers of at least 3mm in diameter; coexisting systemic disease affecting the esophagus (e.g. scleroderma); recent radiation to the esophagus, or caustic or physicochemical trauma to the esophagus; evidence of ZES, esophageal varices, symptomatic pancreatobiliary tract disease, cholecystitis, rheumatoid arthritis, lupus or malignancy; clinically significant abnormal laboratory, biochemical or hematological values or disease; evidence of current alcohol or drug abuse; or required long-term use of ulcerogenic drugs, including NSAIDs, systemic corticosteroids or greater than 325mg/day of aspirin. Patients receiving therapy with PPI or H2RA discontinued use of the antisecretory agent 2 weeks, or within 1 day, respectively, of study initiation, pregnant or lactating patients. |
| **23** | Kahrilas 2000 | NR | US | Multicenter | Patients with confirmed reflux esophagitis (LA grades A-D). | Patients were who tested positive for H pylori at serology screening to eliminate a potentially confounding variable in assessing the efficacy of study medications. Final determination of H pylori status was made by evaluation of the gastric biopsy samples obtained from the antrum and corpus. Patients found to be H pylori positive based on the subsequent biopsy results were allowed to remain in the study. Patients with any bleeding disorder or signs of GI bleeding within 3 days prior to randomization, or had a history of gastric or esophageal surgery, evidence of ZES, primary motility disorders, esophageal stricture, Barrett's esophagus (>3 cm), evidence of upper GI malignancy, severe concomitant disease, or if they were pregnant or lactating, received either PPI therapy within 28 days of the baseline visit or an H2RA on a daily basis during the 2 weeks prior to baseline endoscopy, patients taking NSAIDs or other concomitant medication that might affect the interpretation of the treatment outcome (e.g. diazepam, quinidine, Dilantin, warfarin, anticholinergics, prostaglandin analogs, or sucralfate), and those with a known hypersensitivity to omeprazole or aluminum/magnesium hydroxide (Gelusil). |
| **24** | Kahrilas 2007 | NCT00206245 | US, Canada, France, Germany, Norway, UK, Finland, Italy, Sweden, Denmark | Multicenter | Men and women aged 18-70 years with reflux symptoms and LA A-D reflux esophagitis at baseline endoscopy and required to have a minimum 6-month history of heartburn as their main GERD symptom and experience at least 4 days with episodes of heartburn of at least moderate intensity (ie, discomfort sufficient to cause interference with normal activities) during the 7 days before randomisation. | Patients with current or historical evidence of esophageal stricture; primary esophageal motility disorder; systemic sclerosis; IBS (upper abdominal discomfort or pain that in the opinion of the investigator is likely to be due to IBS or fulfilling 2 or more of the criteria: relieved by defecation, associated with change in frequency of stools, associated with change in appearance of stools); inflammatory bowel disease; ZES; gastric ulcer, duodenal ulcer, or duodenal erosions within the last 3 months. Subjects were also excluded if they had abnormalities of serum chemistry or blood count at the discretion of the site investigator, significant “alarm symptoms,” or a history of drug or alcohol abuse. There was no specified limit for any safety lab variable. However, an intensive monitoring program with repeat testing every third day had to be applied in patients with ALT, AST, or alkaline phosphatase >3× ULN or bilirubin >1.5× ULN, which set the exclusion limit somewhat lower in practical terms. A clinical judgment rather than exact limit was considered appropriate because the clinical significance of abnormal lab tests must be evaluated individually for each patient. Other exclusion criteria included a history of heartburn that was not at least partially relieved within 2 weeks of treatment with PPIs or H2RA and known or suspected hypersensitivity to PPIs or AZD0865. Concomitant therapy with other acid-suppressive therapy, gastroprotective agents, drugs that could alter AZD0865 pharmacokinetics (eg, CYP3A4 inducers or inhibitors), NSAIDs, bisphosphonates, or antineoplastic drugs was not permitted during the study. Patients who used a PPI or had continuous H2RA treatment within the last 14 days of endoscopy and/or between endoscopy and the first dose of study drug. |
| **25** | Kinoshita 2013 | NCT00633932 | Japan | Multicenter | Patients aged 20 years or older diagnosed with LA classification grade A-D esophagitis by upper GI endoscopy. Those untreated or not currently on treatment could be included if a PPI washout period of at least 2 weeks (or for H2RA, the same) was confirmed before randomization. | Patients with conditions like dysphagia, esophageal motility disorders, Barrett’s esophagus, mucosal dysplasia, or gastric/duodenal ulcers; those with a history of upper GI surgery or malignancy; or those with low medication adherence. |
| **26** | Kinoshita 2018 | NCT02463643 | Japan | Multicenter | Adult reflux esophagitis patients assessed as Grade A-D according to the modified LA classification and aged 20 years or older. | Patients with presence of coexisting or previous history of EoE, systemic scleroderma, esophageal varices, esophageal stricture, long-segment Barrett’s esophagus, or high-grade dysplasia; presence of upper GI bleeding, gastric ulcer, or duodenal ulcer within 28 days before the screening period; presence of excessive gastric acid secretion such as due to ZES; presence of a history of esophagus, stomach, or duodenal surgery; receipt of any PPI within 14 days before the screening period; receipt of any drugs for EE within 7 days before the screening period; PPI resistance; presence of severe disease; history of allergy to PPIs; undergoing treatment for a malignant tumor or follow-up duration ≤5 years before the screening period; and pregnancy or nursing. |
| **27** | Korner 2003 | NR | Austria, Germany, Portugal, Switzerland, Netherlands | Multicenter | Male and female patients, aged ≥18 years (for Austria ≥19 years) with endoscopically confirmed reflux esophagitis grade II or III (SM classification) and had suffered from at least moderate heartburn during the 3 days prior. | Patients with any other GI disease, i.e. GERD grades other than II or III, florid peptic ulcer, history of ZES, pyloric stenosis, esophageal or gastric surgery, and severe diseases of other body systems. Patients were excluded if they took PPI, H2RA, prokinetics for more than 10 days, systemic glucocorticosteroids, anti-inflammatory drugs for more than 3 consecutive days or had had PPI-based triple therapy during the previous 28 days, intake of sucralfate during the 3 days prior to study start. |
| **28** | Kovacs 2002 | NR | US | Multicenter | Mmen and non-pregnant women, aged ≥ 18 years, who had endoscopically demonstrated EE, grade 2 or greater on the HD scale, and at least one symptom typical of reflux (night-time or daytime heartburn, or regurgitation) on at least four of the past 7 days. | Patients with Barrett’s esophagus >3 cm in length and/or high-grade dysplasia; peptic ulcers; other upper GI disorders including primary esophageal motility disorders; scleroderma; chronic use of glucocorticoids or NSAIDs other than daily low-dose aspirin, had taken therapeutic doses of H2RA within 2 weeks and other PPI within 1 month of the study medication, or had previously failed treatment with another PPI or H2RA. |
| **29** | Labenz 2005 | SH-NEG-0008 | 14 countries | Multicenter | Patients with photographically documented EE (confirmed by endoscopy and graded using the LA grading system) within 7 days prior to enrolment; history of GERD symptoms for at least 6 months immediately prior to enrolment; heartburn for at least 4 days out of the last 7 days before enrolment, with an overall severity of moderate or severe. | Patients with other significant upper GI disorders (including ZES, gastric or duodenal ulcer, esophageal stricture, history of dysplasia in Barrett’s esophagus); intake of medication liable to affect the outcome of the study (including NSAIDs); pregnancy, childbearing potential (unless taking suitable precautions) or lactation; alcohol and/or drug abuse; PPI use within 2 weeks prior to the first endoscopy. |
| **30** | Laine 2011 | NCT00658528  NCT00658775 | 19 and 21 countries | Multicenter | Men and nonpregnant, nonlactating women aged 18–75 years, with a history of GERD symptoms (e.g. heartburn, regurgitation) for at least 3 months before screening, heartburn at least 2 days/week for ≥1 month before screening endoscopy and moderate-to-severe erosive GERD (LA grade C or D) at screening endoscopy. | Patients with positive UBT for H pylori performed in the month before screening endoscopy; current or history of esophageal motility disorders, Barrett’s esophagus, esophageal strictures, or esophagitis due to etiology other than GERD; a history of upper GI surgery (except simple suturing of an ulcer); ZES or other acid hypersecretory conditions; and current gastric or duodenal ulcer. Patients were not allowed to use PPIs, H2RAs, or prokinetics within 2 weeks before the screening endoscopy or during the treatment phase of the study. |
| **31** | Laine 2023 | NCT04124926 | US and Europe | Multicenter | Adults found at endoscopy to have erosive esophagitis. as confirmed by blinded central reading of endoscopic photographs. | Patients with H pylori infection (patients underwent 13C-UBT during screening) and Barrett’s esophagus, COVID-19. |
| **32** | Laursen 1995 | NR | Denmark | Single center | Outpatients >18 years old with symptoms of gastroesophageal reflux for more than 2. The presence of Barrett’s esophagus was allowed, and so was a benign stricture, provided that it could be balloon-dilated to at least 18 mm. | Patients who are in pregnancy or lactation; concomitant treatment with H2RA, prokinetic agents, sucralfate, alginic acid, or antacids other than trial antacid from the day of the qualifying endoscopy; treatment with any investigational drug within the previous month; esophageal abnormality as part of a systemic disease or infection; ongoing esophageal bleeding; history of esophagogastric surgery except for simple closure of a perforated ulcer; concurrent severe cardiac, renal, or hepatic disease; suspected or confirmed malignancy; concurrent peptic gastric or duodenal ulcer; clinically significant abnormalities in the preentry clinical assessments or laboratory screening; or any condition with suspected association of poor compliance. |
| **33** | Lee 2019 | NCT03006874 | South Korea | Multicenter | Male of female patients aged ≥20 years with endoscopically confirmed EE (LA Classification Grades A-D). | Patients with complications associated with esophagitis, active gastric or duodenal ulcer, GI bleeding, ZES, malignancy, AIDS, hepatitis, EoE, a history of acid-lowering surgery, previous esophageal or gastric surgery, malignancy within 5 years prior to enrolment, primary esophageal motility disorders, IBS, IBD or any of the following abnormal laboratory test values at the screening (BUN and serum Cr level >1.5x ULN; total bilirubin level and serum levels of ALT, AST, ALP and GGT >2x ULN). Pregnant and lactating subjects and those who needed treatment with NSAIDs or surgery requiring hospitalisation. |
| **34** | Lee 2022 | NCT03736369 | South Korea | Multicenter | Male or female patients (20-75 years old) with EE (LA Classification Grades A to D) confirmed by endoscopy at the same institution within 14 days of study treatment initiation. | Patients with Barrett’s esophagus (>3 cm); esophageal stricture; active peptic ulcers; ulcer-related stenosis; GI bleeding; EoE; ZES; IBD; IBS; pancreatitis; psychiatric disorders; AIDS; viral hepatitis; history of gastric acid suppression surgery; significant morbidities in the cardiovascular, respiratory, hepatic, renal, neurologic, endocrine, hematologic, and urologic systems; history of malignancies within five years; drug or alcohol abuse; and hypersensitivity to drugs containing active constituents of esomeprazole or other similar drugs, those who had abnormal laboratory values, including ALT, AST, ALP, GGT, total bilirubin, Cr, and BUN >2x ULN, and women with child-bearing potential. |
| **35** | Lightdale 2006 | SH-QBE-0017/Study 174 | US | Multicenter | Patients aged 18-75 years, with EE confirmed by EGD, and were men or nonpregnant, nonlactating women who were postmenopausal, surgically sterilized, or using an acceptable form of birth control before and throughout the study. All women of childbearing potential had a negative pregnancy test at baseline. | Patients with a positive H pylori serology test at screening; any bleeding disorder or signs of GI bleeding at the time of the screening EGD or within 3 days before randomization; a history of gastric or esophageal surgery, except for simple closure of perforated ulcer; current or historical evidence (within 3 months) of ZES; primary esophageal motility disorders; esophageal stricture; or any serious medical condition including Barrett’s esophagus (>3 cm) or known dysplasia in the esophagus; had used a PPI within 28 days before the baseline visit or an H2RA daily during the 2 weeks before the baseline EGD (occasional use less than daily was permitted) or required an H2RA for the duration of the study; a known hypersensitivity to any component of esomeprazole (Nexium), omeprazole (Prilosec), or antacid (Gelusil) tablets; use of any other investigational compound within 28 days of starting study medication; and previous participation in this study or another clinical study of esomeprazole. |
| **36** | Lim 2023 | NCT04080726 | South Korea | Multicenter | Male and female patients aged 19–75 years with endoscopically confirmed EE based on the LA Classification (grades A–D) and had to experience symptoms of acid regurgitation and/or heartburn 7 days prior to the screening visit. | Patients who had taken PPI and H2RA within 2 weeks before the screening endoscopy, with active gastric or duodenal ulcer, GI bleeding, primary esophageal motility disorders, ZES, IBD, malignancy, and endoscopic Barrett’s esophagus more than 3 cm or esophageal dysplasia, had undergone previous major abdominal surgery, and with abnormal laboratory findings at the screening (MDRD eGFR≤59 mL/min/1.73 m2 or serum Cr>2.0 mg/dL; serum levels of ALT or AST >3x ULN); pregnant or lactating women and those requiring hospitalization for surgery. The patients were not allowed to use any concomitant medications that could affect the evaluation of efficacy, including PPIs, H2RA, antidepressants, antipsychotics, or antianxiety drugs. |
| **37** | Mee 1996 | NR | UK and Ireland | Multicenter | Patients aged between 18 and 80 years, with endoscopically proven reflux esophagitis (SM grades I-IV) and a recent history of at least mild heartburn. | Patients with Barrett's esophagus and/or esophageal ulcer, concomitant peptic ulcer, major co-existent disease, pregnant or lactating women and those who had taken a H2RA within 3 days of trial entry or a PPI within 7 days of trial entry. |
| **38** | Moraes-Filho 2014 | NCT01132638 | Brazil | Multicenter | Adult outpatients (aged 18–70 years) with heartburn or regurgitation at least twice a week for approximately 4– 8 weeks in the previous 3 months and with an endoscopic diagnosis of EE (LA grade A–D). | Patients with any other GI disease, including Barrett’s esophagus, peptic ulcer, ZES, and pyloric stenosis; a history of previous surgery to reduce acid secretion or other surgeries of the upper GI tract (except polypectomy and cholecystectomy); obstructive esophageal strictures, Schatzki ring, esophageal diverticulum, esophageal varices, achalasia or hiatal hernia ≥3 cm on IBD; with severe neurological or psychiatric disorders, hematological disorders, or any other clinically significant medical condition, hepatic or renal dysfunction/disease, clinically significant changes in laboratory parameters, or a history of alcohol or drug abuse within the previous 6 months, pregnant or breastfeeding women or women of childbearing age not using effective contraception if they had used PPIs within 10 days of study commencement; PPI-based triple therapy for eradication of H pylori within the previous 28 days; H2RA, sucralfate or prokinetic agents for 7 days prior to starting the study; or systemic glucocorticoids and/or NSAIDS; including cyclooxygenase-2 (COX-2) inhibitors for more than 3 consecutive days/week within 28 days of the start of the study (except acetylsalicylic acid up to 163 mg/day). |
| **39** | Moretzsohn 2002 | NR | Brazil | NR | Patients age between 18 and 80 years; GERD symptoms particularly heartburn and regurgitation; endoscopic confirmation of non-confluent erosions in the distal portion of the esophagus up to five days before. | Patients who are pregnant or nursing women; previous gastric and/or esophageal surgery; systemic disease associated with GERD; use of PPI and/or PPI antagonists and/or H2RA in the last 20 days. |
| **40** | Mossner 1995 | NR | Germany | Multicenter | Male or female, aged at least 18 years and had reflux esophagitis, grade II or III according to SM, endoscopically diagnosed within 3 days of starting study medication and had at least one of the following key symptoms of GERD: acid regurgitation without nausea, heartburn, or pain on swallowing. | Patients with peptic ulcer, reflux esophagitis grades I or IV, a history of ZES, or patients who had had previous surgery of the esophagus or GI tract; treatment with substituted benzimidazoles in the 30 days prior to study entry, as was concomitant therapy with any drugs whose absorption was pH-dependent or drugs which could interact with substituted benzimidazoles; severe concomitant disease, pregnancy, lactation, lack of reliable contraception in women of child-bearing age, and clinically relevant deviations from the normal range in screening laboratory studies. |
| **41** | Mulder 1996 | NR | Netherlands | Multicenter | Patients aged 18 years or older presenting with reflux esophagitis grade II, III or IVa according to the classification of SM. | Patients with a bleeding ulcer, a stricture requiring more than one dilatation, Barrett’s esophagus without erosions and ulcers above the Barrett segment (grade IVb), coexisting duodenal or gastric ulcer, ZES, concurrent malignant disease (except basal cell carcinoma), previous gastrectomy, vagotomy or operation on the esophagogastric junction; with evidence of uncontrolled, clinically significant cardiovascular, pulmonary, renal, hepatobiliary, pancreatic, metabolic, neurological, endocrine or other system disease; use of a PPI or any investigational drug within 4 weeks of initiating study treatment, as was evidence of current alcohol abuse, illicit drug use, drug abuse, demonstrated intolerance for PPIs, ongoing treatment with any other anti-ulcer medication, corticosteroids and anticoagulants; requiring constant TCAs, reserpine, phenytoin, β-mimetic drugs, cholinergic and anti-cholinergic drugs, although acute treatment for some days was accepted in the month preceding inclusion and during the course of the trial; pregnant or lactating women. |
| **42** | Oh 2025 | NCT05443984 | South Korea | Multicenter | Male or female patients aged 19 years or older with endoscopically confirmed EE (LA Classification Grades A–D). | Patients with complications associated with esophagitis, active gastric or duodenal ulcer, GI bleeding, ZES, hepatitis, EoE, a history of acid‐lowering or gastric surgery, malignancy within 5 years before enrollment, primary esophageal motility disorders, IBS, IBD, and abnormal laboratory test values at the screening (total bilirubin level and serum levels of ALT, GGT >2 ULN and eGFR <70 mL/min/1.73 m2; positive serology for Hepatitis B and C, HIV); pregnant or lactating, those requiring treatment with NSAIDs or hospitalization requiring surgery. |
| **43** | Okanobu 2021 | H29-609-2 | Japan | Single center | Patients aged ≥20 years with GERD symptoms and endoscopically confirmed EE. | Patients treated with H2RA, PPI, and PCAB within 2 weeks before the study; a history of GI resection or vagotomy, esophageal stenosis, achalasia, gastric or duodenal ulcer, IBD, or other serious diseases (hepatic, renal, respiratory, or cardiac disease), pregnancy and malignancy. |
| **44** | Pace 2005 | NR | Italy | Multicenter | Male and/or female outpatients aged ≥18 years; presence of esophagitis of grades I–III (according to the four-degree SM classification) at an endoscopy during the last 7 days prior to inclusion in the trial; a minimum heartburn score of 2 for both frequency and intensity at daytime and/or nighttime; a history of at least 3 months of esophagitis-like symptoms and heartburn for at least 3 days in each of the 2 weeks prior to inclusion. | Patients with esophagitis of infectious origin or caused by exogenous acid or alkaline substances; grade IV esophagitis according to SM; ZES; presence of an active gastroduodenal ulcer or previous esophageal, gastric or biliary surgery (including vagotomy); primary esophageal motility disorders; recent treatment with PPIs (within 2 weeks) and previous (for more than five consecutive days in the 2 weeks prior to trial entry) or concomitant therapy with H2RA, prokinetics, anticholinergics or mucosal protective agents; pregnancy or breast-feeding female; severe liver and/or renal disease, end-stage heart or lung disease; cancer or HIV infection; daily use of NSAIDs; alcoholism or drug abuse. |
| **45** | Petite 1991 | NR | France | Multicenter | Hospitalized or ambulatory patients presenting with symptoms of GERD and on endoscopy showed EE lesions stage 2, 3, or 4 according to SM classification and who had not received any H2RA, prostaglandin derivative, sucralfate, omeprazole, anticholinergics, or cisapride within 14 days. | Patients <18 years, pregnant or breastfeeding women, women of childbearing age without contraception, with a weight deviating more than 15% from ideal weight, serious cardiac, renal, or hepatic disease, gastrectomy or vagotomy, caustic esophagitis, associated gastric or duodenal ulcer, gastric cancer. |
| **46** | Petite 1995 | NR | France | Multicenter | Patients with symptoms of GERD and presence of a stage 2, 3, or 4 esophagitis according to the SM classification during endoscopic examination. | Patients <18 years, whose weight deviated by >15% from ideal values, pregnant or breastfeeding women, women in the reproductive age without effective contraception and patients who received H2RA, prostaglandin analog, sucralfate, omeprazole, antisecretory agents or cisapride in the 14 days preceding the study. |
| **47** | Pilotto 2007 | NR | Italy | Single center | Patients age 65 years or over and endoscopic diagnosis of esophagitis grade I to IV according to the SM classification. | Patients with history of ZES, pyloric stenosis, previous surgery of the esophagus and/or GI tract (except for appendectomy and cholecystectomy), and GI malignancy; had received antacids, sucralfate, prokinetics, H2RA, and/or PPI for more than 7 days in the four weeks prior to the start of the study. |
| **48** | Richter 2000 | NR | US | Multicenter | Men and nonpregnant women ≥18 year of age who had endoscopically demonstrated EE, grade 2 or greater on the Hetzel-Dent scale, and at least one of the symptoms typical for GERD (nighttime or daytime heartburn, or acid regurgitation) on ≥4 of the past 7 days before enrollment. | Patients with Barrett’s esophagus ≥3 cm in length, high-grade dysplasia, peptic ulcers, gastroparesis, or previous gastric or esophageal surgery; use of promotility agents, H2RA within 2 weeks, or other PPI within 1 month before participating in the study. |
| **49** | Richter 2001 | NR | US | Multicenter | Male and female adults aged between 18 and 75 years with photographically documented EE confirmed by EGD and graded according to the LA classification within 1 week before randomization to treatment. Female patients were required to be nonpregnant, nonlactating, postmenopausal, surgically sterilized, or using a medically acceptable form of birth control. | Patients who tested positive for H pylori during serological screening, any bleeding disorder or signs of GI bleeding at the time of the baseline EGD or within 3 days before randomization, history of gastric or esophageal surgery, current or historical evidence of ZES, primary esophageal motility disorders, esophageal stricture, endoscopic Barrett's esophagus (>3 cm) or significant dysplastic changes in the esophagus, duodenal or gastric ulcer, IBD, upper GI malignancy, unstable diabetes mellitus, or other severe concomitant disease. |
| **50** | Robinson 1995 | NR | US | Multicenter | Patients with EE resistant to H2RA therapy, had to have symptoms and esophagitis despite having received at least two different H2RAs, at standard therapeutic disease or higher, during the 12 weeks immediately preceding study enrollment. | Patients <18 years old, not used any potentially ulcerogenic drugs, including NSAIDs or aspirin, chronically within one month prior to the screening endoscopy, or to have received PPI or investigational drugs, including lansoprazole, within 12 weeks. |
| **51** | Schmitt 2006 | SH-QBE-0016/Study 173 | US | Multicenter | Patients, aged 18 to 75 years (inclusive), with EE, confirmed by EGD using the LA classification within 1 week before study randomization. Women were required to be nonpregnant, not lactating, postmenopausal, surgically sterile, or using an acceptable form of birth control. | Patients with positive for H pylori by serology at screening; any bleeding disorder or signs of GI bleeding detected at the time of the screening EGD or within 3 days before randomization; and a history of gastric or esophageal surgery, except for simple closure of perforated ulcer; a history (current or within 3 months) of ZES, primary esophageal motility disorder, esophageal stricture, or any other serious medical condition, including cancer and Barrett’s esophagus (>3 cm by endoscopy). |
| **52** | Sharma 2009 | NCT00251693 NCT00251719 | US and non-US | Multicenter | Adult patients (aged ≥18 years) with endoscopically confirmed EE at screening. Endoscopy was performed by qualified endoscopists to evaluate the presence and severity of EE based on LA Classification grades A–D during the screening period. | Patients who tested positive for H pylori based on the result of the CLOtest (urease test; Kimberly-Clark, Roswell, GA, USA), which was mandatory in all countries outside North America and was performed in the US and Canada only for patients with EE who tested H pylori positive by finger stick or serology at screening, women who were pregnant or lactating and patients who needed to use prescription or nonprescription PPI or H2RA during screening and throughout the study. Patients also were excluded if they used NSAIDs chronically (>12 doses ⁄ month); had coexisting diseases that affected the esophagus, including Barrett’s esophagus with or without dysplastic changes, ZES, esophageal strictures or a history of dilatation of esophageal strictures other than a Schatzki’s ring; had either a history of active gastric or duodenal ulcers within 4 weeks of the first dose of study drug or had acute upper GI hemorrhage within 4 weeks of the screening endoscopy. |
| **53** | Sharma 2025 | NCT05055128 | US and Europe | Multicenter | Patients aged 18–75 years, and body mass index ≥ 18 and ≤ 40 kg/m2 at screening, with endoscopically confirmed EE with either (i) LA grade C or D, or (ii) LA grade A or B together with a documented history of ≥ 8 weeks of PPI therapy plus at least partial symptom response. | NR |
| **54** | Shin 2025 | NCT05267743 | South Korea | Multicenter | Individuals aged 18-80 years with endoscopically confirmed EE. | Patients with inability to undergo upper endoscopy; positive H pylori test; inability to maintain a daily symptom diary; presence of peptic stricture, esophageal varices, Barrett's esophagus, EoE, active peptic ulcer, or bleeding during screening endoscopy; previous surgery or plans to undergo a surgery that could affect gastric acid secretion (i.e., upper gastrectomy and vagotomy); diagnosis of functional dyspepsia, primary esophageal motility disorder, IBD within 3 months before enrollment; history of malignancy within 3 years before enrollment; pregnancy or lactation; use of any PPI 14 days before enrollment; use of any drugs related to GERD treatment (i.e., H2RA, mucoprotectants, prokinetics, antacids) more than twice within 1 week before enrollment; use of HIV protease inhibitors (i.e., atazanavir, nelfinavir) or medications containing rilpivirine; clinically significant abnormal values in screening tests including ALT, AST, ALP, GGT, and total bilirubin levels >2x UNL or BUN and Cr levels >1.5x UNL; history of ZES or gastric acid hypersecretion disorders; permission for continuous use of NSAIDs during the study period; low-dose aspirin (no more than 100 mg/day) to prevent cardiovascular or cerebrovascular events; clinically significant hepatic, renal, cardiovascular, respiratory, endocrine, or central nervous system disorders; and known hypersensitivity to PPIs and PCABs. |
| **55** | Song 2012 | NR | China | Single center | Patients diagnosed with reflux esophagitis based on both outpatient and inpatient settings with at least one typical symptom (heartburn, acid reflux, etc.) and endoscopically confirmed mucosal breaks diagnosed as LA classification grade A-D; aged 20-70 years. | Patients with esophageal or gastric malignancy; severe systemic diseases; ZES or Barrett's esophagus; PPI use within 2 weeks; pregnancy or lactation. |
| **56** | Sontag 1992 | NR | US | Multicenter | Patients aged between 18 and 83 years with symptoms of GERD (such as heartburn) and endoscopic evidence of esophagitis of grade 2 or greater and no history of taking therapeutic doses of H2RA for more than 2 weeks. | Patients with no GERD symptoms; an established primary esophageal motility disorder; esophageal stricture or esophagitis associated with scleroderma; active gastric, pyloric channel or duodenal ulcer; concurrent serious disease, suggested or confirmed malignancy, or clinically significant abnormal laboratory values; concurrent need for NSAIDs, corticosteroids, anticoagulants, anticholinergics, antidepressants, salicylates, or motility drugs of if any dose of antireflux drugs had been taken within 1 week before inclusion. |
| **57** | Uemura 2024 | NCT02679508 | Japan | Multicenter | Patients aged 20 years or older, had EE diagnosed via endoscopy using the LA Classification Grading System (LA grades A–D) at the start of treatment, and were negative H pylori infection (determined by UBT and histological evaluation of the gastric mucosa by Giemsa staining). | Patients who had treatment with PPIs or vonoprazan in the 4 weeks before the healing phase; history of H pylori eradication, allergy, or hypersensitivity to PPIs; and clinically apparent hepatic impairment; pregnant/breastfeeding/ planning to become pregnant, or who had malignancy. |
| **58** | van Rensburg 1996 | NR | South Africa | Multicenter | Outpatients of both sexes over 18 years of age with at least one of the three characteristic symptoms of reflux esophagitis: acid regurgitation, heartburn or odynophagia and have endoscopic evidence of stage II or III reflux esophagitis (SM classification). | Patients with peptic ulcer disease, or complications thereof, as well as those with reflux esophagitis stages I or IV (Barrett’s esophagus, esophageal stricture); with a history of ZES, alcohol or drug abuse, previous surgery of the GI tract (with the exception of appendectomy and cholecystectomy) and the simultaneous intake of glucocorticoids, NSAIDs or drugs known to stimulate reflux (e.g. calcium antagonists, phenothiazines, spasmolytics, nitrates); with severe concomitant diseases (for example of the renal, hepatic or cardiovascular systems) and breastfeeding or pregnant women, as well as women of child-bearing potential without effective contraception (intra-uterinary device, oral contraceptives or injectable hormonal contraception for at least 3 months). |
| **59** | Vcev 1997 | NR | Croatia | Multicenter | Patients presenting with grade I and II esophagitis (SM criteria). | Patients with active peptic ulcer disease, IBD, malignant disease of any organ or previous surgery of the upper GI tract or active biliary or pancreatic disease; treatment with H2RA, omeprazole, sucralfate or prokinetic or anticholinergic drugs during the last 14 days before endoscopy, as was the ongoing treatment with NSAIDs or glucocorticosteroids. |
| **60** | Vcev 1999 | NR | Croatia | Multicenter | Patients presenting with grade I and II esophagitis (SM criteria). | Patients with active peptic ulcer disease, IBD, malignant disease of any organ or previous surgery of the upper GI tract or active biliary or pancreatic disease; treatment with H2RA, omeprazole, sucralfate or prokinetic or anticholinergic drugs during the last 14 days before endoscopy, as was the ongoing treatment with NSAIDs or glucocorticosteroids. |
| **61** | Vcev 2006 | NR | Croatia | Multicenter | Patients with a history of GERD symptoms for at least 6 months immediately prior to enrolment, confirmed by endoscopy and graded using the LA grading system. | Patients with other significant upper GI disorders (including ZES, gastric or duodenal ulcer, esophageal stricture, history of dysplasia in Barrett's esophagus); intake of medication liable to affect the outcome of the study (including NSAIDs); pregnancy, childbearing potential (unless taking suitable precautions) or lactation; alcohol and/or drug abuse; PPI use within 4 weeks prior to the first endoscopy. |
| **62** | Xiao 2020 | NCT02388737 | China, South Korea, Taiwan, Malaysia | Multicenter | Patiets with endoscopically confirmed EE (LA classification grades A-D) within 14 days of randomisation and aged ≥18 years. | Patients with prior exposure to vonoprazan or lansoprazole within 84 days of the observation phase, or exposure to vonoprazan at any time in a previous clinical trial or as a therapeutic agent; hypersensitivity or allergy to vonoprazan, its excipients or to PPIs; significant history of central nervous system, cardiovascular, pulmonary, hepatic, renal, metabolic, GI, urological, endocrine or hematological disease; presence of comorbidities or medical or surgical history that could affect the esophagus; acute upper GI bleeding or gastric or duodenal ulcer within 30 days of the observation phase; history of or treatment for malignancy within 5 years of visit 1; and Cr >2 mg/dL (>177 μmol/L), or ALT, AST or total bilirubin greater than the ULN at the start of the observation phase. |
| **63** | Xue 2016 | NCT01107938 | NR | Multicenter | Patients age 18–70 years (male and female individuals), one of the typical symptoms of heartburn, reflux, confirmed diagnosis of erosive reflux esophagitis by endoscopic examinations 5 days before being selected, according to the LA system. | Patients with cancerous ulcer and failure to exclude cancerous ulcer (excluding moderate to severe dysplasia); peptic ulcer; history of esophageal or gastric surgery; esophageal or gastric varices; ZES; serious complications, such as esophageal stenosis or Barrett’s esophagus; other serious diseases, such as Crohn’s disease and ulcerative colitis; serious heart, liver, lung, kidney, blood, endocrine system diseases; pregnant or lactating women; use of a PPI 5 days before the selection or continuous use of a PPI for more than 3 days within 2 weeks of the study; enrolled in other clinical trials within 3 months of the study; allergy to ilaprazole, esomeprazole, and benzimidazole; alcoholism, drug addiction or other condition inappropriate to clinical drug trials. |
| **64** | Xue 2018 | NCT02860624 | China | Multicenter | Patients aged 18 to 70 years (males and females), with at least one typical reflux symptoms of heartburn, reflux; patients who had confirmed diagnosis of erosive reflux esophagitis by endoscopic examinations 5 days before being enrollment into the study; according to the LA Classification. | Patients having cancerous ulcer, but failed to exclude cancer of ulcer (excluding moderate to severe dysplasia); peptic ulcer; history of esophageal or gastric surgery; esophageal or gastric varices; ZES; have serious complications, such as esophageal stenosis or Barrett's esophagus; other serious diseases, such as Crohn's disease and ulcerative colitis; serious heart, liver, lung, kidney, blood, endocrine system diseases; pregnant or lactating women; using PPIs 5 days before the selection or continuous use of PPIs for>3 days within 2 weeks; enrolled in other clinical trials within 3 months; allergic to Ilaparazole, esomeprazole and benzimidazole; alcoholism, drug addiction or other inappropriate criteria to drug clinical trials. |
| **65** | Zheng 2009 | NR | China | Multicenter | Patients with endoscopically proven reflux esophagitis. | Patients with active peptic ulcer, upper GI cancers, malignant diseases of other organs, severe cardiac, hepatic, or renal diseases, anemia (hemoglobin concentration <10 g/dL), or who were pregnant and/or lactating, |
| **66** | Zhu 2024 | NCT03615677 | China | Multicenter | Patients ≥18 years old, experienced heartburn and symptoms within 7 days prior to screening with at least one of the following symptom assessments in severity and frequency: mild heartburn symptoms lasting at least 2 days and accompanied by reflux symptoms, mild reflux symptoms lasting at least 2 days and accompanied by heartburn symptoms, moderate or above heartburn symptoms lasting for at least 1 day and accompanied by reflux symptoms, or moderate or above reflux symptoms lasting for at least 1 day and accompanied by heartburn symptoms, and diagnosed with EE of LA classification grades A to D by upper endoscopy within 14 days prior to starting study treatment. | Patients with inability to undergo upper endoscopy, history of psychological disorder, possible GI malignancy, malignancies, achalasia, secondary esophageal motility, IBS, IBD, ZES, or EoE, or was taking strong or moderate inhibitors or inducers of the cytochrome metabolizing enzyme CYP3A4 within 4 weeks before the study. |
| **67** | Zhu 2025 | NCT04531475 | China | Multicenter | Male or female patients aged between 18 and 75 years with endoscopically diagnosed EE from LA Grades A to D. | Patients receiving X842 capsules or other PCAB drugs in previous clinical trials. |
| **68** | Zhuang 2024 | NCT05813561 | China | Multicenter | Subjects had endoscopically confirmed EE (LA classification grades A–D) within 7 days of randomization and aged between 18 and 75 years old. | Patients with Barrett’s esophagus (>3 cm); esophageal stricture; active peptic ulcers; ulcer-related stenosis; GI bleeding; EoE; ZES; IBD; IBS; pancreatitis; psychiatric disorders; AIDS; viral hepatitis; significant history of central nervous system, cardiovascular, pulmonary, hepatic, renal, metabolic, GI, urological, endocrine, or hematological disease; presence of comorbidities or medical or surgical history that could affect the esophagus; history of malignancies within 5 years; drug or alcohol abuse; and allergy to fexuprazan, esomeprazole, or other benzimidazole compounds; drug use for GERD within 7 days prior to randomization (e.g. PPIs, PCABs, H2RA, mucosal protective agents, prokinetics, or traditional Chinese medicinal products); with abnormal laboratory values, such as level of ALT, AST, or total bilirubin ≥2 times of ULN; and level of Cr ≥1.5x ULN at screening; female patients with childbearing potential who did not consent to appropriate contraceptive methods use during the study. |

**Supplementary File 5. Baseline Characteristics of Participants Included in Randomized Controlled Trials That Evaluating Potassium-Competitive Acid Blocker and Proton Pump Inhibitor for Maintenance of Healing of Erosive Esophagitis**

**Supplementary File 6. Eligibility Criteria of Included Randomized Controlled Trials Evaluating Potassium-Competitive Acid Blocker and Proton Pump Inhibitor for Maintenance of Healing of Erosive Esophagitis**

| **No** | **Author (Year)** | **Trial Registration** | **Country** | **Center** | **Inclusion Criteria** | **Exclusion Criteria** |
| --- | --- | --- | --- | --- | --- | --- |
| **1** | Ashida 2016 | NCT01452776 | Japan | Multicenter | Patients with healed EE were re-randomized into the long-term study for maintenance therapy. | Patients with complications associated with esophagus, acute upper GI bleeding; history of hypersecretion disorders; surgery or treatment affecting GERD; serious organ disorders; requiring surgery; history of drug abuse; AIDS or hepatitis; history of malignancy; abnormal laboratory test values; pregnant female subjects; concomitant use of medicines affecting the study. |
| **2** | Ashida 2018 | NCT01459367 | Japan | Multicenter | Male or female outpatients aged ≥20 years, who presented with endoscopically confirmed healed EE (no mucosal breaks) after up to 8 weeks of treatment with vonoprazan 20 mg once daily. | Patients with esophageal complications (e.g., EoE, esophageal varices, scleroderma, infection, esophageal stenosis); acute upper GI bleeding; gastric or duodenal ulcer characterized by mucosal defects; hypersecretion disorders, such as ZES; serious neurologic, cardiovascular, pulmonary, hepatic [ALT or AST >2.5× ULN], renal (serum Cr >2mg/dL), metabolic, Gl, urologic, endocrinologic, or hematologic disorders; need for surgery; history of drug (including alcohol) abuse; HIV or hepatitis; history of malignancy; and pregnancy or lactation in females. |
| **3** | Baldi 2002 | NR | Italy | Multicenter | Patients with endoscopic diagnoses of SM grades I–III reflux esophagitis in whom esophageal lesions had healed were admitted to a 6-month maintenance therapy period. Patients on PPI therapy for less than 15 days were submitted to a 7-day washout period before enrollment. | Patients <18 or >70 years or pregnant or breastfeeding; if there was presence of systemic disease or viral or fungal infection, or if they had ingested caustics; with gastric or duodenal ulcer; esophageal varices; esophageal peptic stenosis; Barrett’s esophagus; previous upper digestive surgery; treatment with drugs interfering with gastric acidity (sucralfate, antiacids, bismuth, H2RA, and prostaglandins) or that modify upper digestive motility (antiemetics, prokinetic agents); corticosteroid and/or anticoagulant treatment during the study; continuous or regular treatment with NSAID, phenytoin, warfarin, tricyclic antidepressants, -mimetics, and anticholinergics (patients with treatment lasting up to 15 days before enrollment and during the course of the study were also admitted); diagnosis or suspicion of cancer, ZES, or PPI intolerance; and chronic alcohol or drug abuse. |
| **4** | Bardhan 1998 | NR | UK | Multicenter | Patients with erosive esophagitis of grade 2 or greater (but excluding strictures), proved by endoscopy, and disease healed at 12 or 24 weeks | Patients with concomitant duodenal or gastric ulcers; age <18; pregnancy, lactation, or women planning pregnancy; esophagitis unresponsive to at least three months treatment with H2RA (cimetidine >1.6 g daily or ranitidine >0.45 g); suspicion of upper GI malignancy; significant associated cardiovascular, renal, or liver disease; alcoholism; concomitant phenytoin or warfarin treatments; or known poor compliance. |
| **5** | Bate 1995 | NR | UK and Ireland | Multicenter | Age 18-80 years with minimum of three months' history of symptoms of GERD and SM Grade 2-4 reflux esophagitis on endoscopy. | Patients with esophageal varices, esophageal stricture, upper GI hemorrhage, peptic ulcer, past upper GI surgery or vagotomy. |
| **6** | Birbara 2000 | NR | US | Multicenter | Male and female outpatients of ≥18 years of age, have been diagnosed with endoscopically demonstrable erosive or ulcerative GERD within 90 days. At baseline, endoscopy was required to show no erosive or ulcerative lesions. Female patient were not of childbearing potential or were using an approved method of contraception. | Patients with esophageal ring stricture or esophagitis secondary to systemic events (e.g. scleroderma / ingested irritants), primary esophageal motility disorder, endoscopic evidence of erosive or ulcerative esophagitis, history of definitive acid-lowering surgery or previous esophageal or gastric surgery other than simple closure or perforation, esophageal or gastric varices, or pyloric stenosis that precluded passage of an endoscope, undergoing treatment with anticoagulant, anticholinergics, TCA, motility agent (cisapride, metoclopramide), or antineoplastics, concurrent systemic disorders (renal or hepatic insufficiency), undergoing treatment for cancer, with active peptic ulceration or endoscopic evidence of active GI bleeding, with ZES, those in any condition likely to result in poor compliance, significant abnormalities in laboratory test result, had received any investigational drug other than rabeprazole within 30 days, unable to return for scheduled clinic visit, poor medical or psychiatric risk for therapy with an investigational drug. |
| **7** | Caos 2000 | NR | US | Multicenter | Male and female outpatients >18 years of age diagnosed with erosive or ulcerative GERD and healed as demonstrated by endoscopy within 90 days. Endoscopy requirements at baseline included the absence of active erosions or ulcerations. | Patients with esophageal ring or stricture or esophagitis secondary to systemic events (e.g., scleroderma or ingested irritants); primary esophageal motility disorders; endoscopic evidence of erosive or ulcerative esophagitis; history of definitive acid-lowering surgery or previous esophageal or gastric surgery (except simple closure of perforation); esophageal or gastric varices; or pyloric stenosis that precluded passage of an endoscope; undergoing treatment with anticoagulants, anticholinergics, TCA, motility agents (e.g., cisapride, metoclopramide), or antineoplastics; with concurrent systemic disorders including renal or hepatic insufficiency, as were those undergoing treatment for cancer, those with active peptic ulceration or endoscopic evidence of active GI bleeding, those with ZES, women who were pregnant or lactating, and individuals with any condition likely to result in poor compliance; clinically significant abnormalities in laboratory test results, those who had received any investigational drug other than rabeprazole within 30 days, those unable to return for scheduled clinic visits, and those who were, in the opinion of the investigator, poor medical or psychiatric risks for therapy with an investigational drug. |
| **8** | Carling 1998 | NR | Denmark, Finland and Sweden | Multicenter | Patients of both sexes, aged 18-80 years, with esophagitis Grade 0 or 1 (without erosions) after an initial Grade II, III or IV esophagitis (SM grading scale) treated with lansoprazole 30mg once daily for 8-12 weeks and who reported no severe symptoms of heartburn, regurgitation or dysphagia. | Patients who were pregnant or lactating, had undergone previous upper GI surgery (except simple perforation closure), had a malignancy of the upper GI tract, or severe esophageal disease (e.g. stricture needing dilation, Barrett's esophagus) or other GI disease which was likely to complicate evaluation of esophagitis; taking H2RA, prokinetic agents, PPI, anticholinergics, sucralfate, colloidal bismuth, corticosteroids, anticoagulants or continuous NSAID therapy. |
| **9** | Cho 2023 | NCT04022096 | South Korea | Multicenter | Patients aged 20-75 years with endoscopically confirmed healed EE. | Patients with ZES, GI bleeding, esophageal stricture, pyloric and ulcer stenosis, esophageal gastric varices, Barret’s esophagus >3cm, intractable and digestive ulcer perforation, malignancy on upper GI, hepatic, renal, cardiovascular, respiratory, endocrine or central nervous system disorder, antipsychotics, antidepressants, or anxiolytics usage, history of malignancy or psychiatric disorder, current pregnancy or nursing, usage of PPI, H2RA, prokinetic agent, or antacid within 14 days before screening, or persistent use of NSAID or aspirin during study period. |
| **10** | Dent 1994 | NR | Australia | Multicenter | Outpatients aged between 18 and 86 years who had erosive reflux esophagitis of at least grade 2 severity shown endoscopically within the previous seven days. Patients with benign esophageal stricture were also entered provided their stricture was dilated before entry. Patients with Barrett's esophagus were eligible for study provided they had concurrent esophagitis - that is macroscopic erosions or ulceration. | Patients who are in pregnancy or lactation, inadequate contraception, treatment with prokinetic agents or anti-secretory therapy; concurrent peptic ulcer or complications of ulcer disease; GI malignancy; esophagitis resulting from systemic disease, infection, intubation or other mechanical trauma, burns, irradiation or physical deformity; history of esophagogastric surgery other than simple closure of a perforation or failed anti-reflux surgery without myotomy, vagotomy or a drainage procedure; ongoing upper GI hemorrhage (bleeding associated with peptic mucosal damage had to be controlled adequately before entry); concurrent or past disease, for example, severe cardiac, hepatic or renal disease; clinically significant unexpected abnormalities in the assessments before entry or laboratory screen variables; any condition known to be associated with poor patient compliance; treatment with any other investigational drug during the four weeks before or after the initial endoscopy. |
| **11** | Devault 2006 | D9612L00048/Study 325 | US | Multicenter | Patients 18-75 years of age with healed EE; previously had EE documented by EDG and had heartburn ≥2 days per week during the screening period. | Patients with other GI complications or bleeding disorders or other diseases or conditions that could affect study participation. |
| **12** | Escourrou 1999 | NR | Belgium, France, Italy and Netherlands | Multicenter | Patients with completely healed esophagitis who initially had endoscopically diagnosed GERD grade II or III (SM classification) and were enrolled into a short-term study to heal the acute reflux esophagitis. | Patients with a peptic ulcer and ulcer complications, intake of supportive medication for the management of GERD during the study, regular and continued intake of glucocorticoids, ulcerogenic medications such as NSAIDs, or simultaneous intake of drugs whose absorption is pH-dependent, such as ketoconazole. Pregnant and nursing women, women of child-bearing age without reliable contraception. |
| **13** | Goh 2007 | NR | Europe, Asia, South America | Multicenter | Outpatients 18 years or older with endoscopically confirmed GERD (LA grades A–D) and underwent treatment with pantoprazole 40mg o.d., completed healing (defined as the absence of esophagitis, and ‘no’ or ‘mild’ heartburn and acid regurgitation). | Patients with known history of ZES or other gastric hypersecretory conditions, pyloric stenosis, acute peptic ulcer and ulcer complications, endoscopically negative symptomatic GERD, obstructive esophageal strictures, Barrett’s esophagus and severe diseases of other body systems; pregnant or nursing women and women of child-bearing age. |
| **14** | Gough 1996 | NR | UK and New Zealand | Multicenter | All patients had originally been endoscopically diagnosed with grade 2 or 3 esophagitis and had undergone 8 weeks of healing treatment with 30 mg lansoprazole once daily. Patients were considered to be healed, and thus eligible to enter the maintenance phase. | Women who were pregnant or lactating, patients who had ZES, varices, gastric or duodenal ulcers, known gastric malignancy, scleroderma or active biliary or pancreatic disease. |
| **15** | Hallerback 1994 | NR | Sweden, Norway, Finland | Multicenter | Patients aged 18–80 years with erosive or ulcerative esophagitis grade 2 or more. | Patients with esophageal strictures needing dilatation, concomitant active gastric or duodenal ulcers, and esophageal abnormalities caused by general or systemic diseases, history of esophagogastric surgery other than simple closure of ulcers, malignancy or other diseases with a potential to interfere with the evaluation of the study, pregnancy or lactation, treatment with other investigational drugs within 1 month before inclusion, and drug or alcohol abuse. |
| **16** | Hatlebakk 1997 | NR | Norway | Single center | Men aged 18-80 years or women aged 50-80, or younger if sterilized, with uncomplicated reflux esophagitis diagnosed endoscopically, symptoms were long-lasting and severe enough to justify treatment over an extended period of time. Patients need to be healed and asymptomatic after 12 weeks of PPI treatment. | Patients with peptic stricture, esophageal ulcer, or Barrett's esophagus, gastroduodenal ulcers, hepatobiliary disease, Crohn's disease or previous gastroesophageal surgery, malignant disease or cardiovascular or respiratory disorders if affecting symptom evaluation, needing or having within the last four weeks used H2RA, prokinetics, sucralfate, or drugs with anticholinergic effects, uncooperative or had a history of alcohol or drug abuse. |
| **17** | Howden 2009 | NCT00255164 NCT00255151 | US | Multicenter | Men and women aged ≥18 years with endoscopically proven healed EE after 4- or 8-weeks therapy during 1 of 2 previously described EE healing trials that had compared dexlansoprazole MR 60 and 90 mg with lansoprazole 30 mg. | Patients positive for H pylori by serological assay, women required to be surgically sterile, postmenopausal or using a double-barrier method of birth control, planned surgery during the course of the trial, evidence of uncontrolled systemic disease, need for continuous anticoagulant therapy, use of prescription or nonprescription PPI, H2RA, sucralfate, misoprostol, prokinetics, or non-approved antacids at any time during the trial, use of drugs with significant anticholinergic effects (e.g. TCA) for which a stable dose could not be maintained throughout the trial or chronic use (>12 doses/month) of NSAID, including COX2 inhibitors. |
| **18** | Johnson 2001 | NR | US | Multicenter | Males and nonpregnant, nonlactating females between the ages of 18 and 75 year; had confirmed healing of EE and no record of any SAE related to study medication in the healing study; and were negative for H pylori by histology. Females were to be postmenopausal, surgically sterilized, or using a medically acceptable form of birth control throughout the study. | Patients with endoscopic evidence of either Barrett’s esophagus (>3 cm) or significant dysplastic changes in the esophagus, significant GI disease, any other notable medical conditions or diseases (such as malignancy, pancreatitis, cardiac, pulmonary, liver or renal diseases), or known hypersensitivity to esomeprazole or Gelucil. |
| **19** | Kinoshita 2013 | NCT00634114 | Japan | Multicenter | Patients aged 20 years or older and meeting one of the following: (1) participated in a double-blind comparative study on esomeprazole for initial GERD treatment who were endoscopically confirmed to have reflux esophagitis; (2) confirmed by EGD to have Grade A-D esophagitis within 12 weeks before enrollment. | Patients with dysphagia, motility disorders, hypersensitivity syndromes, IBD, ZES, malabsorption, or Barrett's esophagus, gastric or duodenal ulcers confirmed by endoscopy within 12 weeks, history of surgery on the esophagus, stomach, or duodenum, deemed ineligible by the investigators. |
| **20** | Labenz 2005 | NR | 14 countries | Multicenter | Patients aged ≥18 years with no EE confirmed at the final endoscopy (weeks 4 or 8) during the healing phase of the study, free of moderate to severe heartburn and acid regurgitation for the 7 days immediately prior to entry into the maintenance phase and had no dysplasia in the biopsies taken upon endoscopic suspicion of Barrett’s esophagus. | Patients with the presence of other significant upper GI disorders (e.g. gastric or duodenal ulcers, esophageal stricture, ZES or a history of dysplasia in Barrett’s esophagus) and the use of medication liable to affect the outcome of the study (including NSAIDs). |
| **21** | Laine 2023 | NCT04124926 | US and Europe | Multicenter | Adults found at endoscopy to have erosive esophagitis. as confirmed by blinded central reading of endoscopic photographs. | Patients with H pylori infection (patients underwent 13C-UBT during screening) and Barrett’s esophagus, COVID-19. |
| **22** | Lauritsen 2003 | NR | Europe and South Africa | Multicenter | Patients over 18 years of age, with a history of heartburn (with or without acid regurgitation) and with endoscopically verified reflux esophagitis (LA grade A–D), were treated with 4 weeks of esomeprazole, 40 mg od, asymptomatic patients and those with healed esophagitis were randomized. | Patients with a history of GI surgery; evidence of ZES, upper GI malignancy, abnormal absorption or motility disorders; gastric or duodenal ulcer and/or duodenal erosions within the last 3 months; esophageal stricture; Barrett’s esophagus (>3 cm); or any sign indicating serious or malignant disease. Female patients were required to have a negative pregnancy test, be non-lactating or using a medically acceptable form of birth control. In addition, patients using a PPI for >10 days during the 28 days before baseline, patients with contraindications to the study drugs and those requiring continuous concomitant therapy with medication that might affect the interpretation of the treatment outcome [anticholinergics, cisapride, prostaglandin analogues, NSAIDs or aspirin (except for ≤165 mg/day for cardiovascular prophylaxis)], H2RA, prokinetics and H pylori eradication therapy were not allowed during the study period. |
| **23** | Laursen 1995 | NR | Denmark | Single center | Outpatients >18 years old with symptoms of gastroesophageal reflux for more than 2. The presence of Barrett’s esophagus was allowed, and so was a benign stricture, provided that it could be balloon-dilated to at least 18 mm. | Patients who are in pregnancy or lactation; concomitant treatment with H2RA, prokinetic agents, sucralfate, alginic acid, or antacids other than trial antacid from the day of the qualifying endoscopy; treatment with any investigational drug within the previous month; esophageal abnormality as part of a systemic disease or infection; ongoing esophageal bleeding; history of esophagogastric surgery except for simple closure of a perforated ulcer; concurrent severe cardiac, renal, or hepatic disease; suspected or confirmed malignancy; concurrent peptic gastric or duodenal ulcer; clinically significant abnormalities in the preentry clinical assessments or laboratory screening; or any condition with suspected association of poor compliance. |
| **24** | Metz 2003 | NR | US | Multicenter | Men and women ≥18 years of age with endoscopically demonstrated healed EE (grades 1 or 0 on the Hetzel–Dent scale), required to be outpatients or inpatients not confined to bed with a known history of at least one of the symptoms typical of EE (daytime and/or night-time heartburn, acid regurgitation, or dysphagia). be outpatients or inpatients not confined to bed with a known history of at least one of the symptoms typical of erosive esophagitis (daytime and ⁄ or night- time heartburn, acid regurgitation, or dysphagia). | Patients with esophageal strictures, diverticula, varices or Barrett’s esophagus (>3 cm or with high-grade dysplasia), gastric, pyloric channel, or duodenal ulcers and Zollinger– Ellison syndrome or other gastric hypersecretory, any history of clinically significant GI disorders unstable cardiovascular, pulmonary or endocrine disease; renal or hepatic dysfunction, scleroderma, achalasia, malignancy; chronic use of glucocorticoids, NSAIDs, simultaneous use of pH-dependent drugs, or use of drugs that could interact with the study medication; women who were pregnant, breastfeeding or not using medically acceptable birth control measures. |
| **25** | Metz 2009 | NCT00321737 | US and non-US | Multicenter | Adult men and women (aged ≥18 years) who had participated in 1 of 2 EE healing trials and had endoscopically proven healed EE. | Patients with any condition that may have required surgery during the course of the study; use of prescription or nonprescription PPIs, H2RA or sucralfate; long-term use (>12 doses per month) of NSAIDs including cyclooxygenase-2 inhibitors (aspirin £325 mg daily was permitted); use of antacids [except study-supplied Gelusil (US sites) or a similar equivalent approved antacid (non-US sites)]; use of misoprostol or prokinetics; need for continuous anticoagulant therapy; or evidence of uncontrolled systemic disease. |
| **26** | Park 2013 | NR | South Korea | Multicenter | Patients whose esophagitis was healed and were asymptomatic; previously with typical GERD symptoms such as heartburn, regurgitation or epigastric pain for 2 days or more during the last 7 days in the healing phase of the trial if they were 18 to 75 years of age with a history of reflux symptoms for 6 months or longer, and had endoscopically proven EE (Grade A or B using LA classification of esophagitis). | Patients with endoscopically proven Grade C or D esophagitis; presence of another organic lesion on endoscopy such as peptic ulcer or Barrett’s esophagus; past history of gastric or esophageal surgery; signs of GI bleeding at the time of EGD; chronic alcoholism (＞40 g alcohol/day); heavy smoking (＞2 packs/day); pregnancy or lactation; presence of abnormal liver function test (＞2× normal AST and ALT); known hypersensitivity to any component of omeprazole or rabeprazole; and concomitant diseases that might affect the results such as epilepsy or manic-depressive disorder; had received PPI or H2RA within 15 days before the screening date, or had received other medications that might affect the interpretation of the treatment outcome (quinidine, high dose corticosteroid, NSAID, warfarin, anticholinergic, prostaglandin analogue or salicylate [except low dose aspirin]). |
| **27** | Pilotto 2003 | NR | Italy | Multicenter | Patients age of 65 years or over; an endoscopic diagnosis of esophagitis grade I–III according to the SM classification. | Patients with grade IV esophagitis; gastric and/or duodenal ulcer; a history of ZES; pyloric stenosis; previous surgery of the esophagus and/or GI tract (except for appendectomy and cholecystectomy); malignancy and/or any other severe disease; clinically relevant deviations from the normal range in laboratory values; if they had received antacids, sucralfate, prokinetics, H2RA and/or PPI for more than 7 days in the 4 weeks prior to the start of the study. |
| **28** | Plein 2000 | NR | Austria, Germany, Switzerland | Multicenter | Male and female outpatients previously diagnosed with GERD stage II or III (according to the SM classification) and healed after short-term healing study (lasting up to 8 weeks) with either pantoprazole 40mg or omeprazole 20mg. | Patients with the presence of a peptic ulcer or ulcer complications, intake of supportive medication for the management of GERD during the study, regular and continued intake of glucocorticoids, ulcerogenic medications such as NSAIDs, or simultaneous intake of drugs whose absorption is pH-dependent such as ketoconazole; pregnant and nursing women, women of childbearing age without reliable contraception, clinically relevant deviations from the normal range in laboratory. |
| **29** | Richter 2004 | NR | US | Multicenter | Patients with a known history of at least one of the GERD symptoms including heartburn (daytime and/or night-time) or regurgitation within the month prior to EE healing; men and non-pregnant, non-breastfeeding women 18 years of age or older. | Patients with esophageal strictures, diverticulum, varices, or Barrett’s esophagus ≥3 cm or high-grade dysplasia; evidence of gastric, pyloric, or duodenal ulcers or other clinically significant gastric disorders, including history of surgery of the upper esophagus and/or upper GI tract; other medical diseases such as unstable cardiovascular, pulmonary, or endocrine disease, renal or hepatic dysfunction, or clinically significant hematological, neurological, or psychiatric disorders; evidence of scleroderma, achalasia, history of malignancy, ZES, drug or alcohol abuse or HIV-positive status. |
| **30** | Robinson 1996 | NR | US | Multicenter | Patients had completed an earlier study of short-term lansoprazole treatment for EE or had received a diagnosis of EE and had never received lansoprazole therapy. Patients showed endoscopic evidence of grade 2 or higher esophagitis on the modified SM scale before receiving short-term healing treatment. Patients were required to have endoscopic evidence of healing within 7 days of entering the double-blind maintenance phase. | Patients who were younger than 18 years of age. |
| **31** | Sjostedt 2005 | NR | Sweden | Multicenter | Patients with verified erosive reflux esophagitis (LA grades A–D), confirmed by endoscopy within 7 days prior to visit 1, and at least 18 years old; had to have a history of heartburn episodes over at least 6 months and at least 4 days with heartburn episodes during the week prior to visit 1. They were treated for 4-8 weeks until healed. | Patients with a history of esophageal, gastric or duodenal surgery (except for simple closure of an ulcer), or other GI complications such as ZES, gastric/duodenal ulcer (within last 3 months) or clinically significant esophageal stricture requiring intervention. Significant disease of any other system, as did any suspected or confirmed current malignancy; women of childbearing potential were required to maintain effective contraception during the study period. |
| **32** | Sontag 1996 | NR | US | Multicenter | Patients with healed EE. | Patients <18 years old, history of current drug or alcohol abuse, chronic NSAID or corticosteroid use, uncontrolled clinically significant disease, previous gastric resection, or current esophageal stricture requiring dilation. |
| **33** | Sontag 1997 | NR | USA | Multicenter | Patients with healed esophagitis (grade 0 or 1) experienced fewer than 3 consecutive days of severe heartburn during the final week of phase I. | Patients with infectious esophagitis, primary esophageal motor disorders, scleroderma, esophageal stricture, gastric ulcer, pyloric channel ulcer, duodenal ulcer, Barrett's esophagus >3 cm in length, previous gastric or esophageal surgery, or the need for concurrent, potentially complicating medications such as corticosteroids, NSAIDs, aspirin >162 mg daily, or other antiulcer drugs. |
| **34** | Thjodleifsson 2000 | NR | Europe | Multicenter | Men and women age 18 years and older; women if they were not of childbearing potential or if they were using an approved method of contraception (eg, intrauterine device, oral contraceptives or at least one cycle, implant, or barrier device); with a previous diagnosis of erosive GERD healed within 90 days of enrollment, as documented by endoscopy. | Patients with esophageal ring or stricture or esophagitis secondary to systemic events such as scleroderma (patients with Barrett’s esophagus could be included); primary esophageal motility disorders; endoscopic evidence of EE; history of acid-lowering surgery or previous esophageal or gastric surgery (except simple closure of perforation); esophageal or gastric varices; and pyloric stenosis that precluded passage of an endoscope; had received any investigational agent other than rabeprazole within the previous 30 days. |
| **35** | Uemura 2024 | NCT02679508 | Japan | Multicenter | Patients aged 20 years or older, had EE diagnosed via endoscopy using the LA Classification Grading System (LA grades A–D) at the start of treatment, and were negative H pylori infection (determined by UBT and histological evaluation of the gastric mucosa by Giemsa staining) and had healed EE. | Patients who had treatment with PPIs or vonoprazan in the 4 weeks before the healing phase; history of H pylori eradication, allergy, or hypersensitivity to PPIs; and clinically apparent hepatic impairment; pregnant/breastfeeding/ planning to become pregnant, or who had malignancy. |
| **36** | Vakil 2001 | NR | US | Multicenter | Males and non-pregnant, non-lactating females, between the ages of 18 and 75 years inclusive, who had confirmed healing of EE, no record of any serious adverse event related to study medication in the healing study, and who were negative for H pylori by histology. Females were required to be post-menopausal, surgically sterilized, or using a medically acceptable form of birth control. | Patients with endoscopic Barrett's esophagus (>3 cm) or significant dysplastic changes in the esophagus, as were patients with current or historical evidence (within 3 months) of significant GI disease or any other significant medical condition or disease; use of a PPI (other than as part of the protocol of the healing study) within 28 days prior to the baseline visit; and use of an H2RA daily during the 2 weeks prior to the baseline EGD (occasional use less than daily during these 2 weeks was permitted); taking NSAIDs, quinidine, diazepam, diphenylhydantoin, mephenytoin, warfarin, anticholinergics, prostaglandin analogs, antineoplastic agents, salicylates (unless ≤165 mg daily), oral or intravenous steroids, pro-motility drugs, sucralfate, and those with a known hypersensitivity to esomeprazole or aluminum/magnesium hydroxide. |
| **37** | Xiao 2024 | NCT02388737 | Asia | Multicenter | Patients ≥18 years, with endoscopically confirmed EE within 84 days of randomization and endoscopically confirmed EE healing within 14 days of randomization. Patients with ongoing EE identified at screening were required to complete a healing phase, during which they received open-label treatment with lansoprazole 30 mg q.d. for 4–8 weeks. Patients who achieved EE healing in a phase 3 study of vonoprazan vs. lansoprazole were rerandomized. | Patients with any comorbidity that could affect the esophagus; a history of surgical procedures including gastric or duodenal surgery; or development of acute upper GI bleeding, gastric ulcer, or duodenal ulcer within 30 days of the start of screening; if they had a Cr level >2 mg/dL or ALT, AST, or total bilirubin levels greater than ULN. |

**Supplementary File 7. Risk of Bias Assessment According to the Cochrane ROB2 Tool for Randomized Controlled Trials Evaluating the Healing of Erosive Esophagitis**

| **No** | **Author (Year)** | **Randomization** | **Deviations from intended interventions** | **Missing outcome data** | **Measurement of outcome** | **Selection of reported result** | **Overall** |
| --- | --- | --- | --- | --- | --- | --- | --- |
| **1** | Adachi 2003 | Low | Low | Low | Low | Low | **Low** |
| **2** | Ashida 2015 | Low | Low | Some concerns | Low | Low | **Some concerns** |
| **3** | Ashida 2016 | Low | Low | Low | Low | Low | **Low** |
| **4** | Bardhan 1995 | Low | Low | Low | Low | Low | **Low** |
| **5** | Bardhan 2001 | Low | Low | Low | Low | Low | **Low** |
| **6** | Castell 1996 | Low | Low | Low | Low | Low | **Low** |
| **7** | Castell 2002 | Low | Low | Low | Low | Low | **Low** |
| **8** | Chen 2005 | Low | Low | Low | Low | Low | **Low** |
| **9** | Chen 2022 | Low | Low | Low | Low | Low | **Low** |
| **10** | Cho 2012 | Low | Low | Low | Low | High risk | **High risk** |
| **11** | Cloud 1998 | Low | Low | Some concerns | Low | Some concerns | **Some concerns** |
| **12** | Cordova-Villalobos 1996 | Low | Low | Low | Low | Low | **Low** |
| **13** | Corinaldesi 1995 | Low | Low | Some concerns | Low | Low | **Some concerns** |
| **14** | Dekkers 1999 | Low | Low | Low | Low | Low | **Low** |
| **15** | Delchier 2000 | Low | Low | Low | Low | Low | **Low** |
| **16** | Dupas 2001 | Low | Low | Low | Low | Low | **Low** |
| **17** | Earnest 1998 | Low | Low | Low | Low | Low | **Low** |
| **18** | Fennerty 2005 | Low | Low | Low | Low | Low | **Low** |
| **19** | Gillessen 2004 | Some concerns | Low | Some concerns | Low | Some concerns | **Some concerns** |
| **20** | Hatlebakk 1993 | Low | Low | Low | Low | Low | **Low** |
| **21** | Hetzel 1988 | Low | Low | Low | Low | Low | **Low** |
| **22** | Howden 2002 | Low | Low | Low | Low | Low | **Low** |
| **23** | Kahrilas 2000 | Low | Low | Low | Low | Low | **Low** |
| **24** | Kahrilas 2007 | Low | Low | Some concerns | Low | Some concerns | **Some concerns** |
| **25** | Kinoshita 2013 | Low | Low | Low | Low | Low | **Low** |
| **26** | Kinoshita 2018 | Low | Low | Low | Low | Low | **Low** |
| **27** | Korner 2003 | Low | Low | Low | Low | Low | **Low** |
| **28** | Kovacs 2002 | Low | Low | Low | Low | Low | **Low** |
| **29** | Labenz 2005 | Low | Low | Low | Low | Low | **Low** |
| **30** | Laine 2011 | Low | Low | Low | Low | Low | **Low** |
| **31** | Laine 2023 | Low | Low | Low | Low | Low | **Low** |
| **32** | Laursen 1995 | Low | Low | Low | Low | Low | **Low** |
| **33** | Lee 2019 | Low | Low | Low | Low | Low | **Low** |
| **34** | Lee 2022 | Low | Low | Low | Low | Low | **Low** |
| **35** | Lightdale 2006 | Low | Low | Low | Low | Low | **Low** |
| **36** | Lim 2023 | Low | Low | Low | Low | Some concerns | **Some concerns** |
| **37** | Mee 1996 | Low | Low | Some concerns | Low | Some concerns | **Some concerns** |
| **38** | Moraes-Filho 2014 | Low | Low | Some concerns | Low | Low | **Some concerns** |
| **39** | Moretzsohn 2002 | High risk | Low | Low | High risk | Some concerns | **High risk** |
| **40** | Mossner 1995 | Low | Low | Some concerns | Low | Some concerns | **Some concerns** |
| **41** | Mulder 1996 | Low | Low | Low | Low | Low | **Low** |
| **42** | Oh 2025 | Low | Low | Low | Low | Low | **Low** |
| **43** | Okanobu 2021 | Low | Low | Low | Low | Low | **Low** |
| **44** | Pace 2005 | Low | Low | Low | Low | Some concerns | **Some concerns** |
| **45** | Petite 1991 | Low | Low | Low | Low | Low | **Low** |
| **46** | Petite 1995 | Low | Low | Low | Low | Low | **Low** |
| **47** | Pilotto 2007 | Low | Low | Low | Low | Low | **Low** |
| **48** | Richter 2000 | Low | Low | Low | Low | Low | **Low** |
| **49** | Richter 2001 | Low | Low | Low | Low | Low | **Low** |
| **50** | Robinson 1995 | Low | Low | Low | Low | Low | **Low** |
| **51** | Schmitt 2006 | Low | Low | Low | Low | Low | **Low** |
| **52** | Sharma 2009 | Low | Low | Low | Low | Low | **Low** |
| **53** | Sharma 2025 | Low | Low | Low | Low | Low | **Low** |
| **54** | Shin 2025 | Low | Low | Low | Low | Low | **Low** |
| **55** | Song 2012 | Low | Low | Low | Low | High risk | **High risk** |
| **56** | Sontag 1992 | Low | Low | Some concerns | Low | Some concerns | **Some concerns** |
| **57** | Uemura 2024 | Low | Low | Low | Low | Low | **Low** |
| **58** | van Rensburg 1996 | Low | Low | Low | Low | Some concerns | **Some concerns** |
| **59** | Vcev 1997 | Some concerns | Some concerns | Low | Low | Low | **Some concerns** |
| **60** | Vcev 1999 | Some concerns | Some concerns | Low | Low | Low | **Some concerns** |
| **61** | Vcev 2006 | Low | Low | Low | Low | Low | **Low** |
| **62** | Xiao 2020 | Low | Low | Low | Low | Low | **Low** |
| **63** | Xue 2016 | Low | Low | Low | Low | Low | **Low** |
| **64** | Xue 2018 | Low | Low | Low | Low | Low | **Low** |
| **65** | Zheng 2009 | Low | Low | Low | Low | Low | **Low** |
| **66** | Zhu 2024 | Low | Low | Low | Low | Low | **Low** |
| **67** | Zhu 2025 | Low | Low | Some concerns | Low | Some concerns | **Some concerns** |
| **68** | Zhuang 2024 | Low | Low | Low | Low | Low | **Low** |

**Supplementary File 8. Risk of Bias Assessment According to the Cochrane ROB2 Tool for Randomized Controlled Trials Evaluating the Healing of Erosive Esophagitis**

| **No** | **Author (Year)** | **Randomization** | **Deviations from intended interventions** | **Missing outcome data** | **Measurement of outcome** | **Selection of reported result** | **Overall** |
| --- | --- | --- | --- | --- | --- | --- | --- |
| 1 | Ashida 2016 | Low | Low | Low | Low | Low | **Low** |
| 2 | Ashida 2018 | Low | Low | Low | Low | Low | **Low** |
| 3 | Baldi 2002 | Low | Low | Low | Low | Low | **Low** |
| 4 | Bardhan 1998 | Low | Low | Low | Low | Low | **Low** |
| 5 | Bate 1995 | Low | Low | Low | Low | Low | **Low** |
| 6 | Birbara 2000 | Low | Some concerns | High risk | Low | Low | **High risk** |
| 7 | Caos 2000 | Low | Some concerns | High risk | Low | Low | **High risk** |
| 8 | Carling 1998 | Low | Low | Low | Low | Low | **Low** |
| 9 | Cho 2023 | Low | Low | Low | Low | Low | **Low** |
| 10 | Dent 1994 | Low | Low | Low | Low | Low | **Low** |
| 11 | Devault 2006 | Low | Low | Some concerns | Low | Low | **Some concerns** |
| 12 | Escourrou 1999 | Low | Low | Some concerns | Low | Low | **Some concerns** |
| 13 | Goh 2007 | Low | Low | Low | Low | Low | **Low** |
| 14 | Gough 1996 | Low | Low | Low | Low | Low | **Low** |
| 15 | Hallerback 1994 | Low | Low | Some concerns | Low | Some concerns | **Some concerns** |
| 16 | Hatlebakk 1997 | Low | Low | Low | Low | Low | **Low** |
| 17 | Howden 2009 | Low | Low | Low | Low | Low | **Low** |
| 18 | Johnson 2001 | Low | Low | Low | Low | Low | **Low** |
| 19 | Kinoshita 2013 | Low | Low | Low | Low | Low | **Low** |
| 20 | Labenz 2005 | Low | Low | Low | Some concerns | Low | **Some concerns** |
| 21 | Laine 2023 | Low | Low | Low | Low | Low | **Low** |
| 22 | Lauritsen 2003 | Low | Low | Some concerns | Low | Low | **Some concerns** |
| 23 | Laursen 1995 | Low | Low | Low | Low | Low | **Low** |
| 24 | Metz 2003 | Low | Low | Low | Low | Some concerns | **Some concerns** |
| 25 | Metz 2009 | Low | Low | Low | Low | Low | **Low** |
| 26 | Park 2013 | Some concerns | Low | Low | Low | Low | **Some concerns** |
| 27 | Pilotto 2003 | Some concerns | Low | Some concerns | Low | High risk | **High risk** |
| 28 | Plein 2000 | Low | Low | Some concerns | Low | Some concerns | **Some concerns** |
| 29 | Richter 2004 | Low | Low | Low | Low | Low | **Low** |
| 30 | Robinson 1996 | Low | Low | Some concerns | Low | Low | **Some concerns** |
| 31 | Sjostedt 2005 | Low | Some concerns | Low | Low | Some concerns | **Some concerns** |
| 32 | Sontag 1996 | Low | Low | Some concerns | Low | Low | **Some concerns** |
| 33 | Sontag 1997 | Low | Low | Low | Low | Some concerns | **Some concerns** |
| 34 | Thjodleifsson 2000 | Low | Low | Low | Low | Low | **Low** |
| 35 | Uemura 2024 | Low | Low | Low | Low | Low | **Low** |
| 36 | Vakil 2001 | Low | Low | High risk | Low | Some concerns | **High risk** |
| 37 | Xiao 2024 | Low | Low | High risk | Low | Some concerns | **High risk** |

**Supplementary File 9. Comparison-adjusted Funnel Plot for Healing of Erosive Esophagitis at Week 4. Egger’s test p-value = 0.910.**

**Supplementary File 10. League Table for Healing of EE at Week 4.**

**Supplementary Table 11. Comparison-adjusted Funnel Plot for Healing of Erosive Esophagitis at Week 8. Egger’s test p-value = 0.229.**

**Supplementary File 12. League Table for Healing of EE at Week 8.**

**Supplementary File 13. Network Meta-Analysis illustrating the healing rates of potassium-competitive acid blockers (PCAB) and proton pump inhibitors (PPI) at 2 weeks**. All treatments are compared against vonoprazan 20 mg once daily (QD) as the reference standard and ranked according to their P-score. Higher P-scores indicate superior healing efficacy.

**Supplementary File 14. Comparison-adjusted Funnel Plot for Healing of Erosive Esophagitis at Week 2. Egger’s test p-value = 0.921.**

**Supplementary File 15. League Table for Healing of EE at Week 2.**

**Supplementary File 16. Network Meta-Analysis illustrating the healing rates of potassium-competitive acid blockers (PCAB) and proton pump inhibitors (PPI) at (A) 2 weeks, (B) 4 weeks, and (C) 8 weeks for erosive esophagitis patients with Los Angeles Grade A/B equivalent (Mild/Moderate).** All treatments are compared against vonoprazan 20 mg once daily (QD) as the reference standard and ranked according to their P-score. Higher P-scores indicate superior healing efficacy.

**Supplementary File 17. Network Meta-Analysis illustrating the healing rates of potassium-competitive acid blockers (PCAB) and proton pump inhibitors (PPI) at (A) 2 weeks, (B) 4 weeks, and (C) 8 weeks for erosive esophagitis patients with Los Angeles Grade C/D equivalent (Severe).** All treatments are compared against vonoprazan 20 mg once daily (QD) as the reference standard and ranked according to their P-score. Higher P-scores indicate superior healing efficacy.

**Supplementary File 18. Network Meta-Analysis illustrating the healing rates of potassium-competitive acid blockers (PCAB) and proton pump inhibitors (PPI) at (A) 2 weeks, (B) 4 weeks, and (C) 8 weeks for erosive esophagitis patients with Los Angeles Grade B-D equivalent.** All treatments are compared against vonoprazan 20 mg once daily (QD) as the reference standard and ranked according to their P-score. Higher P-scores indicate superior healing efficacy**.**

**Supplementary File 19. Comparison-adjusted Funnel Plot for Any Adverse Events in Randomized Controlled Trials Evaluating Healing of Erosive Esophagitis. Egger’s test p-value = 0.890**

**Supplementary File 20. Comparison-adjusted Funnel Plot for Serious Adverse Events in Randomized Controlled Trials Evaluating Healing of Erosive Esophagitis. Egger’s test p-value = 0.313**

**Supplementary File 21. League Table for Maintenance of Healed EE at 6 Months.**

**Supplementary File 22. Comparison-adjusted Funnel Plot for Maintenance of Healing of Erosive Esophagitis at 6 months. Egger’s test p-value = 0.502.**

**Supplementary File 23. Network Meta-Analysis of maintenance of healing rates of potassium-competitive acid blockers (PCAB) and proton pump inhibitors (PPI) at 6 months based on Los Angeles (A) Grade A/B equivalent, (B) Grade C/D Equivalent, and (C) Grade B-D equivalent**. Vonoprazan 10 mg once daily (QD) is the reference treatment. Treatments are ranked based on their P-scores, where higher scores denote improved rates of non-recurrence.

**Supplementary File 24. League Table for Maintenance of Healed EE at 3 Months.**

**Supplementary File 25. Comparison-adjusted Funnel Plot for Maintenance of Healing of Erosive Esophagitis at 3 months. Egger’s test p-value = 0.657.**

**Supplementary File 26. League Table for Maintenance of Healed EE at 12 Months.**

**Supplementary File 27. Comparison-adjusted Funnel Plot for Maintenance of Healing of Erosive Esophagitis at 12 months. Egger’s test p-value = 0.957.**

**Supplementary File 28. Comparison-adjusted Funnel Plot for Any Adverse Events in Randomized Controlled Trials Evaluating Maintenance of Healing of Erosive Esophagitis. Egger’s test p-value = 0.336.**

**Supplementary File 29. Comparison-adjusted Funnel Plot for Serious Adverse Events in Randomized Controlled Trials Evaluating Maintenance of Healing of Erosive Esophagitis. Egger’s test p-value = 0.409.**
